# Discovery of a novel small secreted protein family with conserved N-terminal IGY motif in Dikarya fungi

**DOI:** 10.1186/1471-2164-15-1151

**Published:** 2014-12-20

**Authors:** Qiang Cheng, Haoran Wang, Bin Xu, Sheng Zhu, Lanxi Hu, Minren Huang

**Affiliations:** Jiangsu Key Laboratory for Poplar Germplasm Enhancement and Variety Improvement, Nanjing Forestry University, Nanjing, 210037 China; College of prataculture science, Nanjing Agricultural University, Nanjing, China

## Abstract

**Background:**

Small secreted proteins (SSPs) are employed by plant pathogenic fungi as essential strategic tools for their successful colonization. SSPs are often species-specific and so far only a few widely phylogenetically distributed SSPs have been identified.

**Results:**

A novel fungal SSP family consisting of 107 members was identified in the poplar tree fungal pathogen *Marssonina brunnea*, which accounts for over 17% of its secretome. We named these proteins IGY proteins (IGYPs) based on the conserved three amino acids at the N-terminus. In spite of overall low sequence similarity among IGYPs; they showed conserved N- and C-terminal motifs and a unified gene structure. By RT-PCR-seq, we analyzed the *IGYP* gene models and validated their expressions as active genes during infection. IGYP homologues were also found in 25 other Dikarya fungal species, all of which shared conserved motifs and the same gene structure. Furthermore, 18 IGYPs from 11 fungi also shared similar genomic contexts. Real-time RT-PCR showed that 8 *MbIGYPs* were highly expressed in the biotrophic stage. Interestingly, transient assay of 12 MbIGYPs showed that the MbIGYP13 protein induced cell death in resistant poplar clones.

**Conclusions:**

In total, 154 IGYPs in 26 fungi of the Dikarya subkingdom were discovered. Gene structure and genomic context analyses indicated that *IGYPs* originated from a common ancestor. In *M. brunnea*, the expansion of highly divergent MbIGYPs possibly is associated with plant-pathogen arms race.

**Electronic supplementary material:**

The online version of this article (doi:10.1186/1471-2164-15-1151) contains supplementary material, which is available to authorized users.

## Background

Fungi are osmotrophic microorganisms, which utilize various secreted proteins to obtain nutrients and adapt to ecological niches [1, 2]. Plant pathogenic fungi secrete diverse groups of small proteins, which have been implicated in the establishment of parasitic relationships. For example, clusters of small secreted protein (SSP) genes in *Ustilago maydis* have been shown to be essential for virulence [3], and comparative genomic analysis of eighteen Dothideomycetes fungi revealed that pathogenic fungi usually have more predicted SSPs compared with their saprotrophic counterparts [4]. Moreover, most characterized fungal effectors are small secreted proteins, which can manipulate the cellular processes of hosts to facilitate infection [5, 6]. Therefore, the identification and analysis of SSPs has been highlighted in genomic studies assessing many plant pathogenic and symbiotic fungi [7–9]. However, as a rule, SSPs are always highly species-specific and lack similarity to known proteins. For example, in the genomes of the rust fungi *Melampsora larici-populina* and *Puccinia graminis* f. sp. *tritici*, 74% and 84% of predicted SSPs are lineage-specific [7]. Therefore, it remains as a challenge to predict the functions of SSPs and discover new effector candidates in non-model fungi.

To date, only very few widely distributed SSPs have been described, despite the continually increasing genome/transcriptome data available for fungi. Examples of widely distributed fungal SSPs include necrosis- and ethylene-inducing-like proteins (NLPs), which can trigger cell death in a wide range of dicotyledonous hosts by inducing plasma membrane leakage [10]. Moreover, NLP homologues are also found in many pathogenic bacteria and oomycetes, with a dramatic expansion of NLPs in oomycetes observed [11]. Other representatives are fungal LysM effectors, which enhance pathogen virulence by suppressing the chitin-triggered immunity of host cells. LysM effectors also occur in nonpathogenic fungi; indeed, a LysM effector of the plant-beneficial fungus *Trichoderma atroviride*was shown to inhibit spore germination of *Trichoderma* spp., implying that LysM effectors have potentially different roles [12, 13]. Cerato-platanins (CPs) are a group of conserved small secreted cysteine-rich proteins found in both Ascomycete and Basidiomycete fungi [14]. CPs are abundant in many fungal secretomes and potentially have different functions [15]. The Ecp2 effector was originally discovered in the apoplast of *Cladosporium fulvum* infected tomato leaves and shown to be indispensable for *C. fulvum* virulence [16]. A recent *in silico* study showed that Ecp2 homologues with conserved Ecp2-domains constitute a superfamily and are widely distributed in the subkingdom Dikarya [17]. Some powdery mildew and rust fungi have effector candidates with a conserved Y/F/WxC motif at the N-terminus of mature proteins [18]. However, Y/F/WxC motifs are not restricted to the N-terminal regions and occur at high frequency in non-secreted proteins of other fungi [7, 19].

The ascomycete *Marssonina brunnea,* which belongs to the order of Helotiales, is a widespread agent of black spot disease of poplar. *M. brunnea* causes defoliation and thus growth reduction of susceptible poplar clones, making it a major constraint on poplar plantation. Unlike other phytopathogens in Helotiales, such as *Sclerotinia sclerotiorum* and *Botrytis cinerea,* which are exemplary necrotrophs with a very wide range of hosts, *M. brunnea* has a hemibiotrophic lifestyle and displays a high degree of host specialization within the *Populus* genus. The availability of genome sequence of a specific form, *M. brunnea* f. sp. *multigermtubi,* provides the opportunity to screen its virulence genes involved in the pathogenesis [20–24].

In a previous study, we identified the “species-specific” SSP MbEcp10 in the secretome of *M. brunnea* [23]. With the rapid advances in fungal genome sequencing, we reassessed MbEcp10 and found a gene family encoding MbEcp10-like proteins in the genomes of *M. brunnea* and other Dikarya fungi. This family is likely to have a common origin and significantly represented in *M. brunnea*. RT-PCR-seq, real-time RT-PCR and transient assay were performed for *M. brunnea MbEcp10-like* gene analysis. Our findings imply that expansion and divergence of *M. brunnea* MbEcp10-like proteins are likely associated with plant-pathogen arms race.

## Results and discussion

### A small secreted protein family in *M. brunnea*

**Figure 1:**
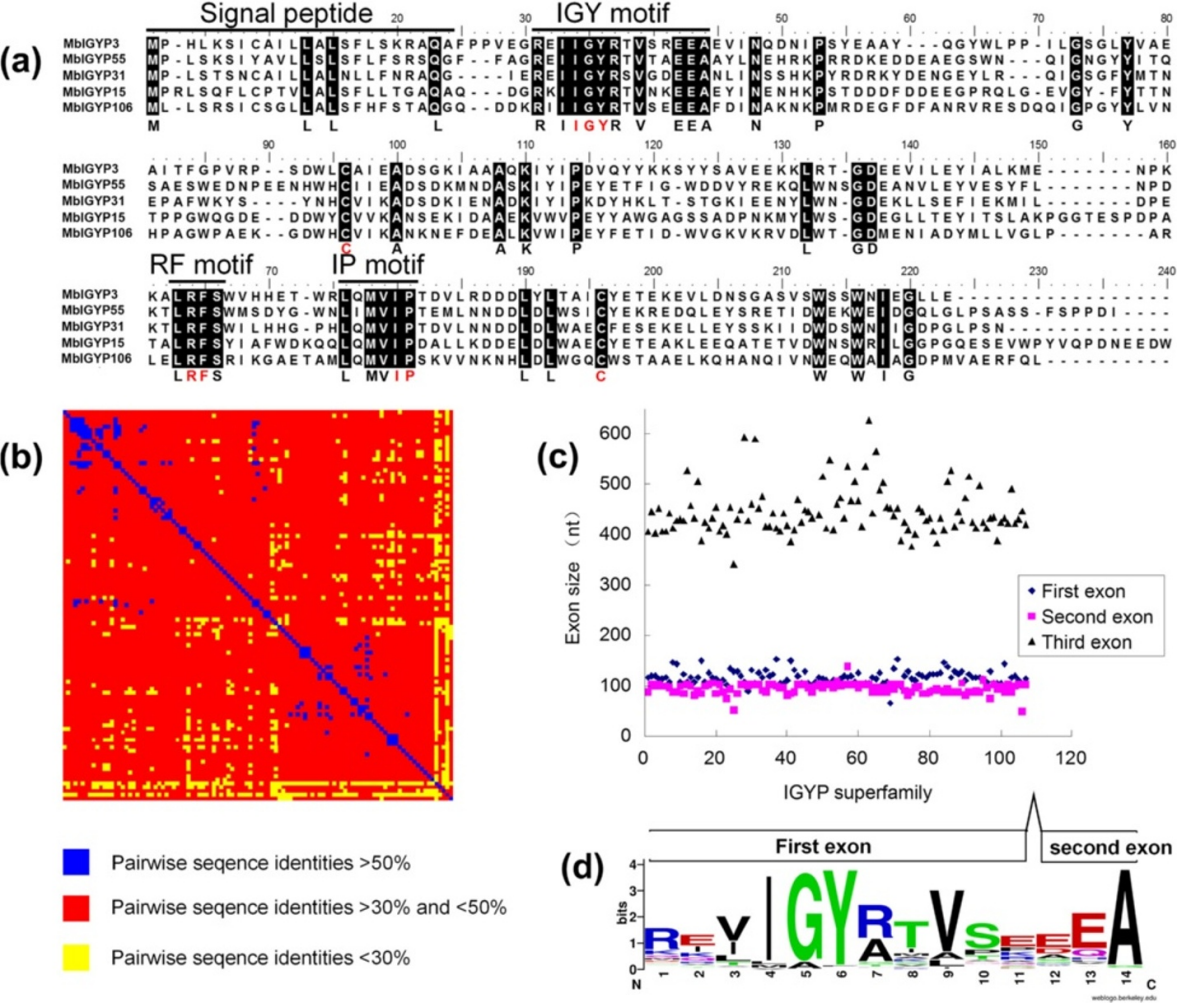
**Characterization of the** ***M. brunnea*** **IGYP family. (a)** Amino acid alignment of five members showing conserved motifs at N- and C-termini. Conserved motifs were overlined. **(b)** Pairwise identity of 107 members of the *M. brunnea* IGYP superfamily. **(c)** Exon size distribution of 100 *M. brunnea IGYPs*. **(d)** Consensus sequence pattern of the IGY motifs (14 amino acids) calculated with WebLogo based on an alignment of the 107 *M. brunnea* IGYPs. 11 amino acids in N-terminus are encoded by the first exon. 3 amino acids in C-terminus are encoded by the second exon.

The MbEcp10 sequence was used to BLAST against a local database of *M. brunnea* predicted proteins, which was downloaded from GenBank [22]. The identity of 94 predicted proteins exceeded 30%, with the best hit reaching 44%. Despite the low overall similarity, all of them were small proteins with obvious signal peptide sequences, and displayed highly similar N-terminal and C-terminal regions (Figure 1a), suggesting the presence of a protein family related to MbEcp10 in *M. brunnea.* Using the recursive BLAST search, 107 MbEcp10-like proteins were found, each with at least one significant BLAST hit (identity >40%) to another MbEcp10-like molecule. The relatedness of a protein family can be illustrated by the pairwise similarity of the protein pairs [25]. As shown in Figure 1b, 93.7% pairwise comparisons between any pair of resultant proteins displayed >30% sequence identity (red and blue regions); for any protein, at least 27 pairwise comparisons with the other 106 proteins showed >30% sequence identity. This set of pairwise relationships defines a fully connected network, indicating that the 107 proteins comprise a single protein family. Meanwhile, extensive sequence divergence among these related proteins was observed that only 1.5% of pair sequences with identities exceeding 50% in pairwise comparison (Figure 1b and Additional file 1).

Beside sequence similarity, these proteins also displayed three obvious common features, indicating that they have a common ancestry:Firstly, most of them were small, secreted proteins, with 105 members consisting of 187-268 aa; only two members had no signal peptide sequence. Secondly, these proteins had conserved motifs and cysteines in the same position. Next to the signal peptide at the N-terminus, they contained a 14–amino acid motif with a 3–amino acid core consisting of two hydrophobic amino acids and one small molecular amino acid in between. This motif was found in 106 members (in 86 members, the core was IGY, for Isoleucine, Glycine, and Tyrosine). Therefore, we named this motif IGY and the MbEcp10-like proteins were called IGYPs (IGY proteins). At the C-terminus, a conserved 5 amino acid motif was found: QMxIP (for Glutamine, Methionine, any hydrophobic amino acid, Isoleucine, and Proline). The two-amino acids IP were the most conserved and the motif was named IP. There was a less conserved motif upstream of the IP motif with the LRFS (for Leucine, Arginine, Phenylalanine, and Serine) sequence, which we named the RF motif. In addition, in the middle and at the C-terminus of most IGYPs (106 IGYPs), there were two conserved cysteine residues (Figure 1a).

Thirdly, the *IGYP* genes shared a similar structure: the open reading frame (ORF) regions of most predicted *IGYPs* (100 *IGYPs*) consisted of three exons. The sizes of the three exons were conserved across the 100 *IGYP* genes. The 5′-terminus of the second exon always encoded the C-terminus of the IGY motif, and had the most conserved size (Figure 1c,d and Additional file 1).

In the genome of *M. brunnea*, 559 genes were predicted as secreted proteins-encoding genes [22], indicating that IGYPs account for about 17% (105/599) of the *M. brunnea* secretome and suggesting that the IGYP family could be pivotal for successful adaption to the ecological niche.

RxLR-dEER double-motifs are the host targeting signals for pathogenic Oomycete RxLR effectors, which are found at the N-terminus of mature proteins [26]. Interestingly, the IGY and RxLR-dEER motifs showed similar features (Figure 1a, d). The N-termini of IGY motifs in 47 MbIGYPs (*M. brunnea* IGYPs) were consistent with the extended RxLR-like motif definition of [R⁄K⁄H]X[L⁄M⁄I⁄F⁄Y⁄W]X [27]. In addition, a total of 79 IGY motifs showed alkaline N-terminus followed by hydrophobic sequence as found in the RxLR-like motif. Moreover, 53 of the 79 IGY motifs had acidic C-terminal, with 2-3 continuous Es (Glutamic acid residues) followed by A (Alanine), similar to the dEER motif that neighbors the RxLR motif. The IGY motifs were also located at the N-termini of mature proteins with 0-17 amino acids from the signal peptides. These similarities between the IGY and RxLR-dEER motifs suggest IGY to be a potential host targeting signal.

### *MbIGYP*gene models were validated by RT-PCR-seq

**Figure 2:**
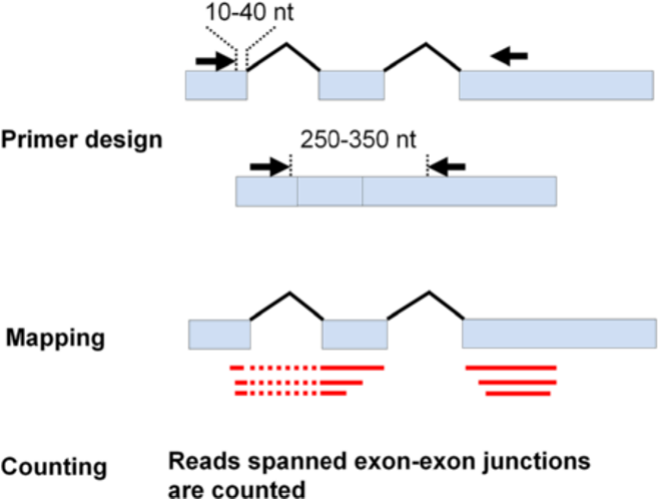
**Principle of primer design and bioinformatics workflow.** Position of primers (black arrow) designed for validating gene models of *MbIGYPs* and sequencing reads (red lines) mapped on targeted exons (light blue rectangles).

RT-PCR-seq is an extremely sensitive method for validating gene models of low-expressed transcripts [28]. The predicted gene structures strongly indicate that *MbIGYPs* originated from the same ancestral gene. However, the use of regular RT-PCR to confirm these structures with mycelia growing in synthetic liquid medium was inefficient (data not shown). Therefore, we tentatively applied the RT-PCR-seq method to test *MbIGYPs*’ gene models with mix samples (i.e. inoculated poplar leaves with *M. brunnea* spores). Samples at 0, 1 and 4 dpi (days after inoculation) were collected and analyzed separately (Additional file 2: Figure S1). The RT-PCR primers were placed in the first and third exons, respectively, and forward primers were closely adjacent to the first exon-exon junction (see Materials and Methods) (Figure 2). With respect to seven *MbIGYPs,* of which the predicted gene models were not composed of three exons, the primer pairs were chosen at approximate regions by alignments with homologous genes.

RT-PCR-seq yielded 3348762, 4694790 and 6187204 sequencing reads from 0, 1 and 4 dpi samples, respectively. A total of 91 *MbIGYPs* had more than 6 sequencing reads spanning the exon-exon junctions. Given the high sensitivity of the RT-PCR-seq method, 14 *MbIGYPs* without reads spanning the exon-exon junctions and 2 *MbIGYPs* with only one read spanning these junctions (Additional file 3) were likely to be inactive pseudogenes. Based on sequencing results, we corrected the splicing sites in 13 wrongly annotated *MbIGYP* gene models, including seven genes previously predicted with inconsistent gene structures, and found that all tested *MbIGYPs* had two introns in sequencing reads for covering regions (Additional files 1 and 3).

The RT-PCR-seq results validated that most *MbIGYPs* (91) were active during the infection process, further confirming that the *MbIGYP* genes share a consistent gene structure.

Because RT-PCR-seq provided deep sequencing at exon-exon junctions, this targeted approach also allowed us to analyze alternative splicing (AS) of *MbIGYP* genes. In the 181 exon-exon junctions covered by sequencing reads, 10 alternative splicing sites for 9 *MbIGYP* genes were found, with each site supported by >10 sequencing reads, which accounted for 5.5% exon-exon junctions of *MbIGYPs*, a slightly lower rate than the average (6.0%) obtained for Ascomycota [29]. However, considering the highly sensitive method used for testing *MbIGYPs* AS, these rates could be far below average. From all AS events, we only found one exon skipping (SE) event in *MbIGYP29*. The other alternative splicing types were either alternative 5′ splice site (A5′SS) or alternative 3′ splice site (A3′ SS) (Table 1).

**Table 1:** **Alternative splicing in** ***MbIGYP*** **genes**

Gene ID	AS type*	AS position	0 dpi	1 dpi	4 dpi	Mature protein size	Splicing manner
			Isoform1 reads/Isoform2 reads	Isoform1 reads/Isoform2 reads	Isoform1 reads/Isoform2 reads	Isoform1/Isoform2	Isoform1/Isoform2
*MbIGYP101*	A3′SS	First intron	4/1	1/3	1742/2730	37 Aa/198 Aa	GT-AG/GT-AG
*MbIGYP23*	A3′SS	First intron	20/10659	29497/79306	6932/24700	12 Aa/169 Aa	GT-AG/GT-AG
*MbIGYP29*	A5′SS and A3′SS and SE	First intron and second intron	0/36189	195/110363	83/46546	153 Aa/186 Aa	CT-AC/GT-AG
*MbIGYP39*	A5′SS and A3′SS	First intron and second intron	0/142719 0/355	351/36850 136/141	9626/177704 54/764	20 Aa/190 Aa	CT-AC/GT-AG
*MbIGYP53*	A3′SS	First intron	0/1	0/7174	1279/6690	42 Aa/39 Aa	GT-AT/GT-AG
*MbIGYP62*	A5′SS	First intron	1/75	4/3	1972/4251	19 Aa/223 Aa	GT-AG/GT-AG
*MbIGYP32*	A5′SS and A3′SS	Second intron	0/0	0/0	28/69	32 Aa/203 Aa	GC-AC/GT-AG
*MbIGYP36*	A5′SS	First intron	2/15	0/460	96/2183	37 Aa/198 Aa	GT-AG/GT-AG
*MbIGYP56*	A3′SS	Second intron	0/74	0/1079	31/726	57 Aa/195 Aa	GT-AG/GT-AG

*A3′SS is alternative 3′ splice site; A5′SS is alternative 5′ splice site; SE is exon skipping.

In addition, non-canonical splicing sites, including CT-AC, GT-AT and GC-AC, were also found in 1 and 4 dpi samples but not in 0 dpi samples, even though these splicing sites were also covered by comparable sequencing reads in pre-infection samples. This result suggested that non-canonical splicing might be related to the fungal infection process (Additional file 2: Figure S2 and Table 1).

### *IGYP*homologues are patchily distributed in the subkingdom Dikarya

**Figure 3:**
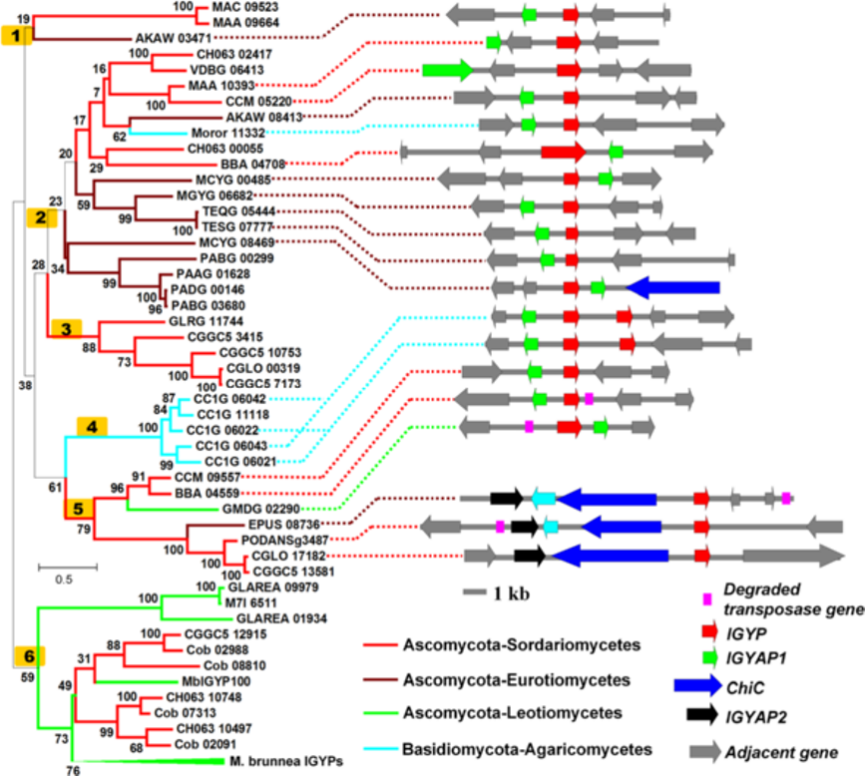
**Phylogeny of IGYPs in Dikarya and genomic context of IGYPs.** The left panel represents the phylogenic tree inferred by the maximum likelihood method with the amino acid sequences of all 154 IGYP homologues in Dikarya (Ascomycota and Basidiomycota). The IGYP homologues are shown with Gene ID, and species names were abbreviated as follows: *Metarhizium acridum* (MAC), *Metarhizium anisopliae* (MAA), *Aspergillus kawachi* (AKAW), *Colletotrichum higginsianum* (CH063), *Verticillium alfalfa* (VDBG), *Cordyceps militaris* (CCM), *Moniliophthora roreri* (Moror), *Beauveria bassiana* (BBA), *Arthroderma otae* (MCYG), *Arthroderma otae* (MGYG), *Trichophyton equinum* (TEQG), *Trichophyton tonsurans* (TESG), *Paracoccidioides brasiliensis*Pb03 (PABG), *Paracoccidioides* . ‘lutzii’ Pb01 (PAAG), *Paracoccidioides brasiliensis* Pb18 (PADG), *Colletotrichum graminicola* (GLRG), *Colletotrichum gloeosporioides* Nara gc5 (CGGC5), *Colletotrichum gloeosporioides* Cg-14 (CGLO), *Coprinopsis cinerea* (CC1G), *Pseudogymnoascus destructans* (GMDG), *Endocarpon pusillum* (EPUS), *Podospora anserine* (PODANSg), *Glarea lozoyensis* (GLAREA), *Glarea lozoyensis* (M7I), and *Colletotrichum orbiculare* (Cob). Species and accession numbers are given in Additional file 4. Numbers with yellow background represent major clades. Numbers adjacent to nodes are bootstrap values. IGYP branches from 4 classes are arranged with different colors. The right panel depicts the genomic context of *IGYPs*. Protein-coding genes adjacent *IGYPs* are shown as colored arrows, denoting transcriptional orientation. The *IGYP* genes are shown as red arrows in the center. Green, blue, and black arrows are conserved cluster members, while grey arrows are non-homologous adjacent genes.

In order to identify IGYP homologues in other species, we searched the public database using the deduced *M. brunnea* IGYP protein sequences*.* A total of 47 homologous proteins were found from 25 fully sequenced fungal species or isolates. Interestingly, IGYP homologues were not confined to a limited phylogenetic fungal branch, but patchily distributed in 4 classes of the subkingdom Dikarya, including Sordariomycetes (Ascomycota), Eurotiomycetes (Ascomycota), Leotiomycetes (Ascomycota), and Agaricomycetes (Basidiomycota). Unlike *M. brunnea*, these fungi only had 1-5 *IGYP* homologous genes without apparent gene expansion (Figure 3 and Additional file 4).

**Figure 4:**
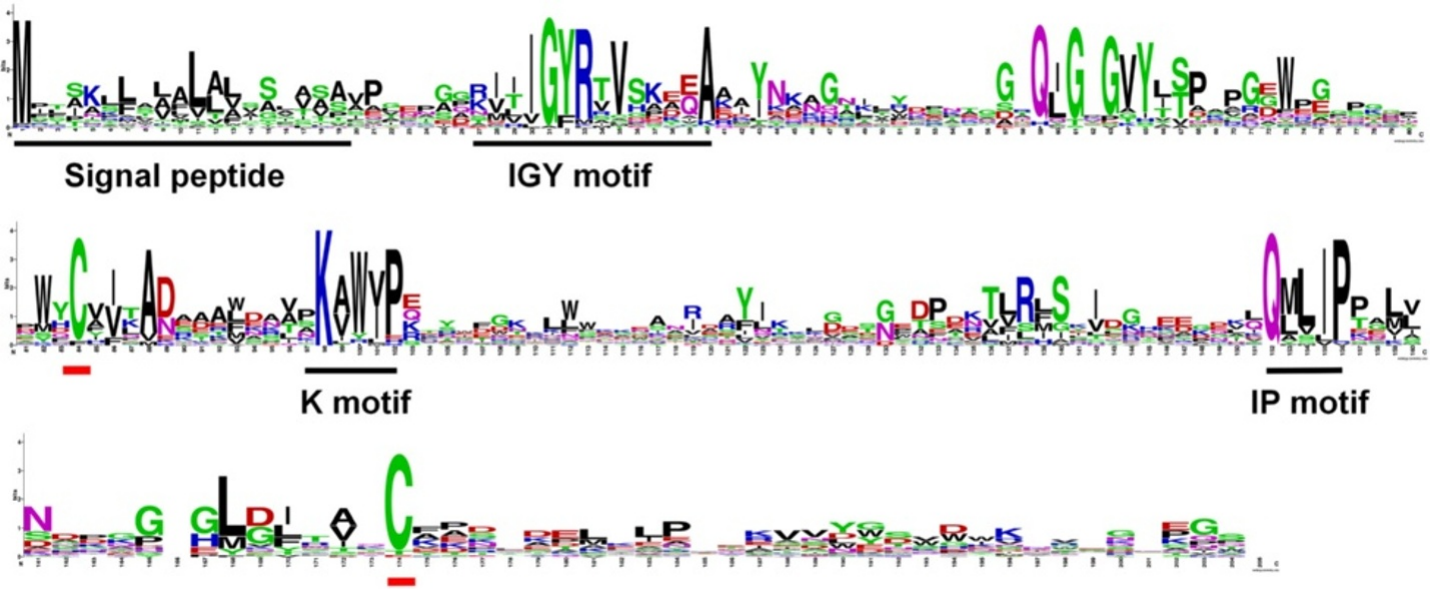
**Consensus sequence pattern of 47 IGYP homologous proteins from 25 fungal species.** The consensus sequence pattern was generated with WebLogo based on alignment of 47 IGYP homologous proteins, of which gaps were removed. Conserved motifs and cysteine residues were underlined.

Although the 47 IGYP homologous proteins showed low similarity (identity <50%) with any MbIGYPs, most of them met the common criteria that define the IGYP family of proteins in *M. brunnea*. First, 40 homologous proteins were of small size (<300 aa), and 44 had signal peptide sequences. Second, 45 of them displayed obvious IGY motifs immediately adjacent to the signal peptides; 22 had conserved QMxIP motif in C-terminus, and 19 had analogous QxxxP (x is any hydrophobic amino acid) motifs. RF motifs were not obvious, but in the middle of IGYP homologues there was a conserved 5-amino acid KxWxP motif (x is Alanine, Valine or Isoleucine), which was less conserved in the 107 MbIGYPs. Mature IGYP homologues had 2-4 cysteine residues, of which two cysteines in the middle and at C-terminus were conserved (Figure 4). Third, the 38 homologous genes consisted of three exons, with the third significantly larger than the first two. The IGY motifs of 44 members were encoded by two exons, and their C-termini were commonly encoded by the 5′-termini of the second exons (Additional file 4).

Figure 3 shows a phylogenetic tree constructed by the maximum likelihood method. Most nodes had low bootstrap support, which is partially due to limited phylogenetic information obtained from small and highly divergent IGYPs. Phylogenetic information is usually limited in small proteins and could be lost by saturated substitution [30]. However, there were still several clades with high bootstrap support, such as IGYPs from *M. brunnea* (MbIGYPs), *Coprinopsis cinerea* (CC1G) and *Paracoccidioides spp.* (PABG, PAAG and PADG), suggesting these proteins diverged recently.

### *IGYPs*have similar genomic contexts

Syntenic groups of homologous sequences can be used to determine the orthology of compared sequences [31]. Thus, we analyzed the genomic contexts of *IGYP* genes by BLAST search. We found that the immediately adjacent locus of 18 *IGYPs* in11 fungi encoded a homologous hypothetical protein, which was named IGYAP1 (IGYP adjacent protein 1) (Figure 3 and Additional file 4). The intervals between the *IGYP* and *IGYAP1* genes ranged from 609 to 3340 bp. Using the BLASTp program to search the NCBI public database, we found that 24 *IGYAP1* homologous genes (*E* value < 0.1) and only 8 genes were not adjacent to the *IGYP* loci. In another case, a chitinase C gene (*ChiC*) and an unknown protein-encoding gene (named as *IGYAP2*) were found adjacent to three *IGYP* genes in three fungus species (*Endocarpon pusillum* (EPUS)*, Podospora anserine* (PODANSg) and *Colletotrichum gloeosporioides* (CGLO) (Figure 3 and Additional file 4). These results indicate significant conservation of the genomic context in some *IGYP* genes across different fungal species. This can be considered as additional evidence supporting a common lineage of some *IGYPs*.

Ascomycota and Basidiomycota diverged from one another at least 400 million years ago (Mya) [32]. It is therefore unexpected that small gene clusters, such as *IGYP* and *IGYAP1* families exist in both Ascomycota and Basidiomycota. This can be hardly explained by complicated gene duplication and loss. Phylogenetic analysis of IGYPs revealed that one clade with high bootstrap value (clade 5, 79% of bootstrap) was highly incongruent with the known phylogenetic relationships of species (Figure 3). In addition, independent analysis of IGYAP1 and ChiC also revealed a similar incongruent phylogenetic relationship between the adjacent neighbors of clade 5 IGYPs (Additional file 2: Figure S3). Moreover, in clade 5, a degraded transposase gene was found neighboring the *IGYP-IGYAP1* clusters of *Beauveria bassiana* and *Pseudogymnoascus destructans* as well as the *IGYP-ChiC-IGYAP2* clusters of *P. anserine* and *E. pusillum* (Figure 3). These results suggest that horizontal gene transfer could be more parsimonious, and constitute the probable explanation for the presence of some *IGYPs* along with its neighbors in phylogenetically-distant fungal species.

### The *IGYP*genes of *M. brunnea*are highly expressed in the biotrophic stage

**Figure 5:**
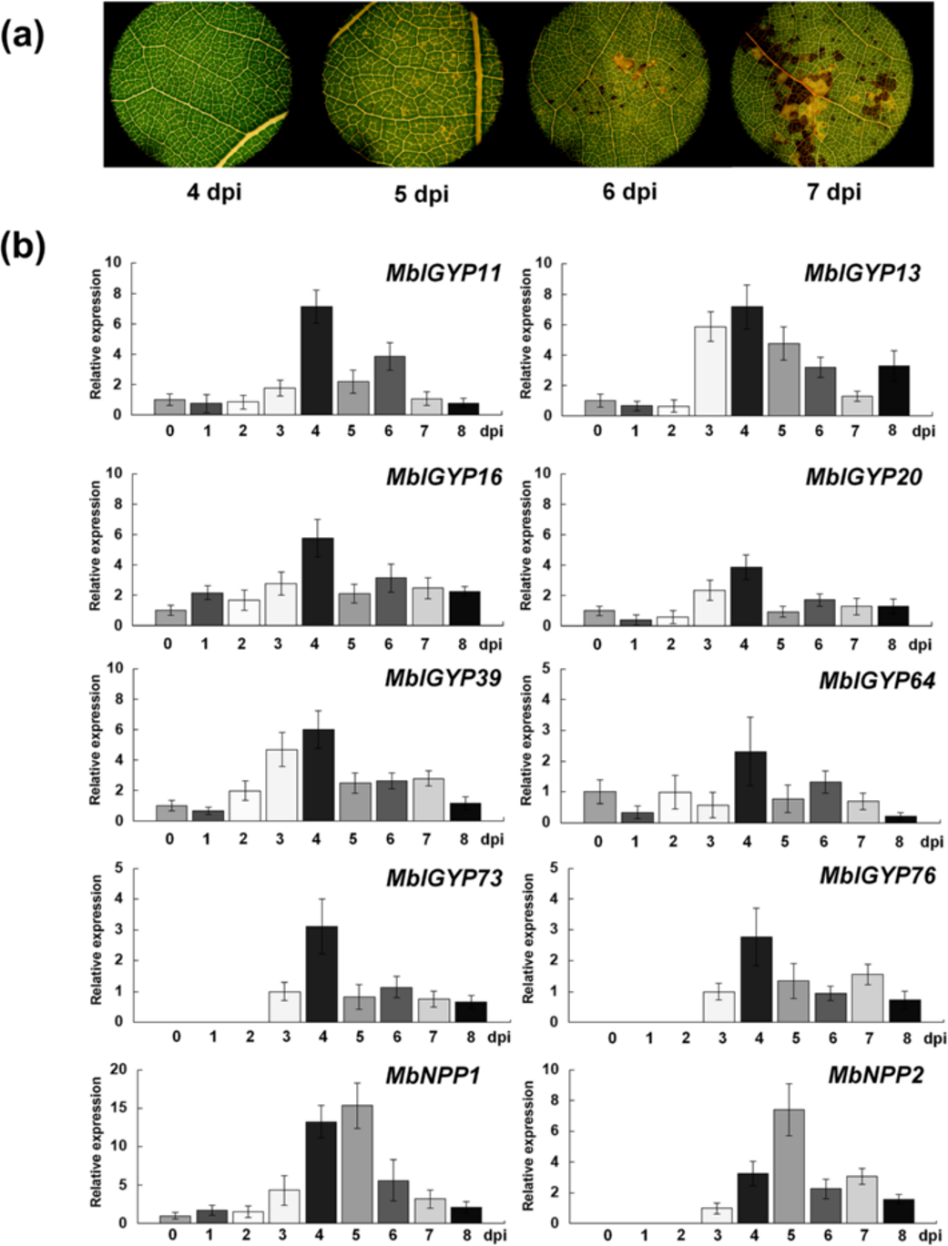
**Expression patterns of** ***MbIGYPs*** **during infection of susceptible poplar clone** ***.*** **(a)** Disease symptom development after inoculation of *M. brunnea* at 4, 5, 6 and 7 dpi. **(b)** *MbIGYP* gene expression patterns analyzed by real-time RT-PCR. For *MbIGYP11*, *13*, *16*, *20*, *39*, *64*, *73, 76, MbNPP1 and MbNPP2*, expression levels were calculated relatively to 0 dpi samples. Because of low expression levels of *MbIGYP73*, *76* and *MbNPP2* at 0-2 dpi, their relative expression levels were calculated based on 3 dpi samples. Bars indicate average expression ± SD of three technical replicates. These experiments were repeated three times with similar results.

*M. brunnea* is a hemibiotrophic fungal pathogen. Upon inoculation of the susceptible poplar clone I-214, no visible symptoms are observed at 0-4 dpi. At 5 dpi, small chlorotic spots can be found on leaf surfaces. At 6 dpi, black spots appear on some chlorotic spots. At 7 dpi, the chlorotic and black spots join into small pieces (Figure 5a).

In order to analyze the gene expression patterns of *MbIGYPs* during infection, eight active *MbIGYP* genes were selected for real-time RT-PCR experiments. Cytotoxic NLP expressions are usually associated with the transition from biotrophic to necrotrophic phase in phytopathogens [33–35]. We chose two *M. brunnea* NLP homologous genes (*MbNPP1* and *MbNPP2*), which are homologous to *BcNep1 and BcNep2* of *Botrytis cinerea* [36], as controls in real-time RT-PCR experiments.

As shown in Figure 5b, seven of the eight *MbIGYP* genes had a uniform expression pattern. From 0 to 2 dpi, *MbIGYP11*, *13*, *16*, *20*, *39*, *73*, and *76* maintained low expression levels without significant induction. At 3 dpi, these seven genes were induced and their expression peaked at 4 dpi, then dramatically declined at 5 dpi. In contrast to the other *MbIGYP* genes, *MbIGYP64* was induced in the early days after infection, and its expression peaked at 4 dpi. A dramatic decrease of *MbIGYP64* expression was also seen at 5 dpi.

On the other hand, two NLP homologous genes showed different expression patterns. Both *MbNPP1* and *MbNPP2* were induced at 3 dpi and their expression levels continued to increase at 4 dpi. At 5 dpi, their expression levels peaked, and dramatically declined at 6 dpi (Figure 5b).

Because the first visible disease symptoms emerging and MbNPP1/2 expression peaks coincided time wise (5 dpi), *M. brunnea* might switch from biotrophic to necrotrophic growth at the fifth day after inoculation. Thus, all tested *MbIGYP* genes achieving their highest expression levels at 4 dpi with a stark decline afterwards suggest that the *MbIGYP* family genes are specifically induced and expressed in the biotrophic stage.

### A *M. brunnea*IGYP protein induces cell death in resistant poplar clones

**Figure 6:**
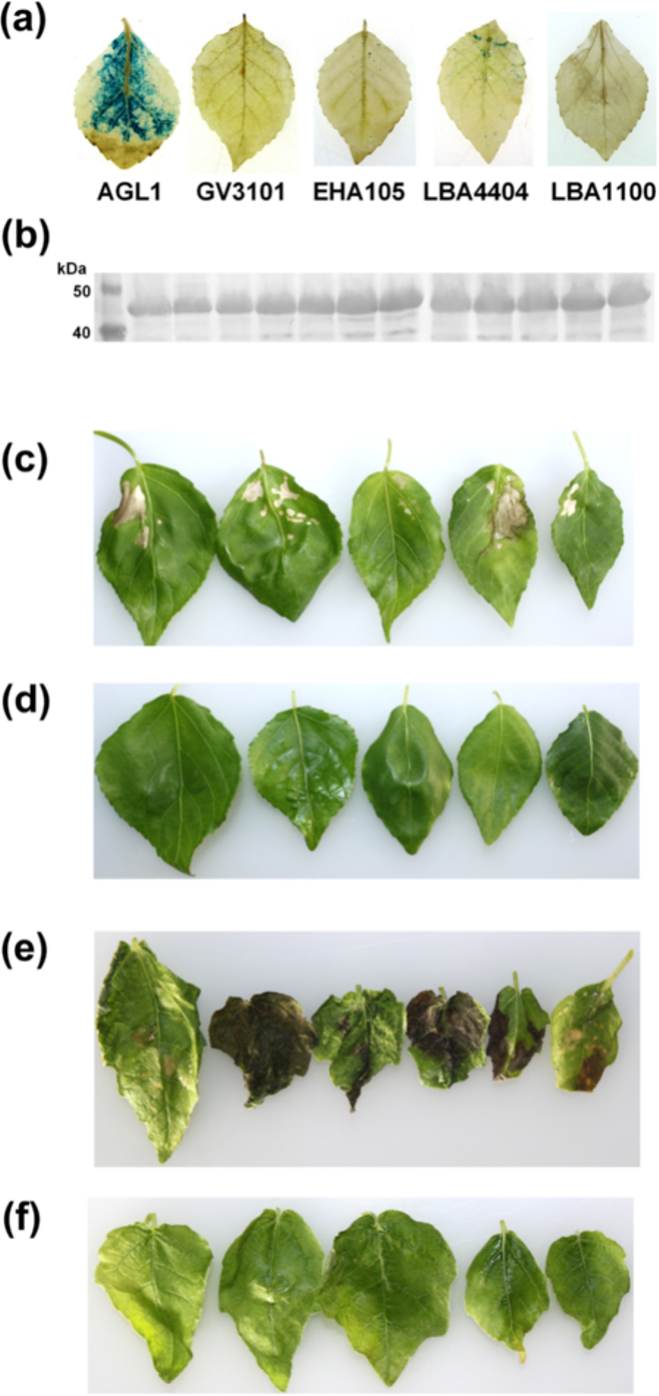
**Transient expression assays by** ***Agrobacterium*** **-mediated vacuum infiltration. (a)** Staining for GUS activities in leaves of NL895 infiltrated with five *Agrobacterium* stains carrying pCambia1305.1. **(b)** Expression of 12 MbIGYPs in NL895 leaves was detected by Western blot using an anti-GFP antibody. From left to right: protein ladder, MbIGYP59, MbIGYP6, MbIGYP13, MbIGYP50, MbIGYP52, MbIGYP10, MbIGYP43, MbIGYP33, MbIGYP71, MbIGYP79, MbIGYP87 and MbIGYP94. **(c)** Cell death symptoms on the resistant poplar clone NL895 at 5 dai with AGL1 harboring *MbIGYP13*. **(d)** NL895 leaves infiltrated with AGL1 containing the empty vector at 5 dai (negative control). **(e)** Necrosis phenotype of non-host *P. tomentosa* leaves at 5 dai after infiltration with AGL1 carrying *MbIGYP13*. **(f)** *P. tomentosa* leaves infiltrated with AGL1 containing the empty vector for 5 days (negative control). Photographs **(c)**-**(f)** show 5-6 leaves collected from a single seedling. Experiments were repeated twice with similar results.

The *M. brunnea*-resistant hybrid poplar clone NL895 is a progeny of *P. deltoides* I-69 and *P. euramericana* I-45, which are resistant and susceptible clones, respectively [37] *.* Therefore, the poplar clone NL895 was selected for transient assays of MbIGYPs. As a first step, five different *Agrobacterium* strains (AGL1, LBA4404, LBA1100, GV3101 and EHA105) were transformed with the pCambia1305.1 vector carrying a β-glucuronidase (*GUS*, *uidA*) gene with an artificial intron. Then, the transformed *Agrobacterium* strains were infiltrated into the tissue cultured plantlets of NL895 by the *Agrobacterium*-mediated vacuum infiltration method [38]. The results of histochemical GUS assays showed that the *Agrobacterium* strain AGL1 produced significantly more intense GUS staining compared with other *Agrobacterium* strains (Figure 6a).

Since the majority of known fungal and Oomycete effector proteins either function directly in the apoplast or translocate into plant cells using pathogen-independent mechanisms, we started with expressing MbIGYPs with signal peptides. The 12 *MbIGYP* cDNAs encoding mature proteins (MbIGYPs-Δsp) were fused in-frame to a *Arabidopsis* PR1 signal peptide coding sequence (PR1sp) by overlapping PCR; such PCR products were cloned into the Gateway-compatible vector PH35GY (containing C-terminal YFP) to generate expression constructs driven by the CaMV 35S promoter (PH35GY-35S-PR1sp-MbIGYPs-Δsp-YFP). The resultant binary vectors were transformed into *Agrobacterium* stain AGL1. Subsequently, the transformed *Agrobacterium* strains were infiltrated into the tissue cultured plantlets of NL895. Exogenous MbIGYP expression at 4 days after infiltration (dai) were confirmed by Western blotting using anti-GFP antibody and the detected molecular weights (about 45-48 kDa) were close to the predicted values (46-51 kDa) for YFP-fusion proteins (Figure 6b). The results of transient assays showed that MbIGYP13 induced cell death which appeared as discrete tissue desiccation on NL895 leaves at 5 dai (Figure 6c). In contrast, other MbIGYPs (not shown) and the negative control (*Agrobacterium* stain carrying empty vector) failed to induce any visible lesions on NL895 leaves (Figure 6d). *P. tomentosa*, a non-host of *M. brunnea*, showed higher expression efficiency than the hybrid poplar NL895 (Additional file 2: Figure S4). Transiently expressing PR1sp-MbIGYP13-Δsp-YFP induced necrosis and crinkling of *P. tomentosa* leaves at 5 dai (Figure 6e). In contrast, no necrotic responses were observed upon agro-infiltration of any other MbIGYPs (not shown) and the negative control (Figure 6f). Collectively, these data showed that among the 12 tested MbIGYPs, only MbIGYP13 induced cell death in poplar, and the cell death response to MbIGYP13 is likely conserved among several resistant *Populus* species.

## Conclusions

In this study, we identified a novel protein family named IGYP in the subkingdom Dikarya based on five criteria: (1) similarity of amino acid sequences, (2) small size proteins with signal peptides, (3) conserved N-terminal and C-terminal motifs, (4) conserved gene structure, and (5) similar genomic context. In total 154 proteins were identified as IGYPs supported by at least three criteria above mentioned. These *IGYPs* are scattered across 4 classes of Ascomycota and Basidiomycota, but may have a single origin.

In *M. brunnea, 107* genes were identified as *IGYPs.* In contrast, only 1-5 copies of *IGYP* homologues can be observed in other 25 fungi. This suggests a large lineage-specific expansion of *IGYP* genes in *M. brunnea.* In addition, phylogenetic analysis of IGYPs showed that all *MbIGYP* sequences except for MbIGYP100 cluster together, formed a species-specific clade in the tree. This large lineage-specific expansion of *MbIGYP* genes is likely the result of recent evolution in *M. brunnea*. Because hemibiotrophic plant pathogens exhibit characteristics of biotrophs in the early stages of infection, avoiding recognition by the plant immune system is essential for early secreted proteins. We speculate that induction of cell death in resistant poplar by a biotrophic stage specific MbIGYP could be a failure case of escaping host surveillance; the expansion of these highly divergent MbIGYPs is possibly associated with plant-pathogen arms race.

## Methods

### Search for IGYP homologues and motif finding

The MbEcp10 protein sequence (ADB23426) was searched against the *M. brunnea* predicted proteins local database using BLASTp. Based on the IGY motif next to the N-terminus and the IP motif in the C-terminus, homologous proteins were picked out manually and removed from the database. Then this set of homologous proteins was also searched against the database using BLASTp until no more proteins with IGY motif could be found. IGYP homologous proteins in other fungi were searched against the GenBank nr database using BLASTp with the *M. brunnea* IGYP protein sequences. Presence of N-terminal IGY motif was set as screening criterion.

Homologous proteins of IGYAP1 were obtained by BLASTp search against the GenBank nr database with the protein sequences of *Cordyceps militaris* and *Coprinopsis cinerea* IGYAP1 (XP_006674752 and XP_001829814, respectively). The cutoff was *E* value < 0.1. Homologous proteins of ChiC were obtained by BLASTp search with the *Colletotrichum gloeosporioides* Cg-14 ChiC protein sequence (EQB44099). Top30 hits were selected for further phylogenetic construct. Homologous proteins of IGYAP2 were obtained by BLASTp search with the *Colletotrichum gloeosporioides* Cg-14 IGYAP2 protein sequence (EQB44100). The cutoff was *E* value < 1e-10^7^.

The predications of the signal peptides and cleavage sites were performed using SignalP4.1 Server [39]. The multi-alignments and pairwise comparisons were performed using Bioedit (v7.2.5) and ClustalW2 server (http://www.ebi.ac.uk/Tools/msa/clustalw2/). Sequence logos in Figures 1 and 4 were produced by WebLogo [40].

### Phylogenetic analysis

The phylogenetic trees of IGYPs, IGYAP1, ChiC and IGYAP2 were constructed with the maximum likelihood method using MEGA 6.0 [41]. The WAG + gamma model was selected, and gaps were partially deleted (30%). The inferred phylogenies were tested by 100 bootstrap replicates.

### Plant material and pathogen infection

The branches of *Populus euramericana* cv. I-214 were cut out in winter and grown in pots at 22°C with a 12-hour photoperiod. The cuttings took root and produced 20-50 leaves after 12 weeks. Leaves were cut off and placed into culture dishes of sterilized 1% water agar with abaxial surface up.

Conidia of *M. brunnea* f.sp. *multigermtubi* were cultured in PDA medium for ten days. The conidia were suspended again in deionized water, adjusted to 10 000 spores/mL and sprayed to the up-side of leaves. Treated leaves were harvested at 0, 1and 4 days post-inoculation (dpi), frozen quickly using liquid nitrogen, and stored at -70°C for RNA isolation.

### RT-PCR-seq

Briefly, the *IGYP* genes were amplified using traditional RT-PCR, and then the PCR products were analyzed by high-throughput sequencing.

#### Primer design

The primers were designed based on the predicted *IGYP* genes. All the primers were 20 bp in length. The forward primers were located in the first exons, and the 3′-terminuses were 10-20 bp from the splicing sites. The reverse primers were located in the third exons. The location of the reverse primers was adjusted to make the lengths of RT-PCR products 250-350 bp. Because the PCR products span two introns, most of the products’ lengths would exceed 400 bp if using the genomic DNA as template (Figure 2 and Additional file 5).

#### RT-PCR-seq

Total RNA was extracted using the RNAprep pure Plant Kit (Tiangen) with on-column DNaseI digestion from samples of 0, 1, 4 dpi. Reverse transcription was conducted with equal amount of total RNA (5 μg) using the SuperScript® III First-Strand Synthesis System (Invitrogen), and the resulting cDNA was diluted 1:3 with nuclease-free water.

Each PCR reaction used 3 μL cDNA. The PCR amplification was conducted using the Hot Start *Taq*DNA polymerase (Takara) in 50 μL volume. The following cycling condition was used: 95°C for 10 min and 38 cycles at 95°C for 15 s, 56°C for 30s and 72°C for 1 min. PCR products of *IGYP* genes in different infection time points were pooled separately, and 1 mL of pooling PCR products were gel-purified to remove >400-bp products and primer dimers and eluted by 200 μL ddH_2_O.

The purified samples were used to build libraries using a TruSeq DNA sample prep kit (Illumina). The TruSeq libraries were sequenced using Illumina HiSeq2000 with 100-bp paired-end reads. Each library produced more than 2 Gb raw data. Low-quality bases (≦Q20), adaptors and short reads (≦50 bp) were removed. The sequences were aligned using TopHat2 software against the gDNA reference sequences of *IGYPs* [42].

#### Analyzing gene model

The alignment results were viewed and analyzed with Tablet program [43]. The criterion for gene model validation was at least 5 reads spanning the exon-exon junctions. The criterion for alternative splicing was more than 10 reads to support the existence of the alternative exon-exon junction, and pair-sequences of these reads were also aligned to the same reference to avoid the influence of heteroduplex.

### Real-time RT-PCR

The inoculation conditions were identical with those for RT-PCR-seq. Treated leaves were sampled at 0, 1, 2, 3, 4, 5, 6, 7 and 8 days. Disease symptoms were observed daily using a stereo microscope (Leica MZ950). RNA extraction and reverse transcription methods were the same as RT-PCR-seq. Specific primers of 24-bp length were designed based on the predicted gene sequence (Additional file 5). *Elongation factor 1-a* was chosen as an internal control gene [23]. Relative expressions were calculated by method. 0 dpi sample expression levels were set to 1 and all subsequent sample expression levels were compared with the 0 dpi samples.

PCR reactions were carried out in a 20-μL reaction system using the FastStart Universal SYBR Green master mix (Roche). Real-time RT-PCR was performed on an Applied Biosystems 7500 Real-time PCR System (Applied Biosystems). The following cycling conditions were used: 50°C for 2 min, 95°C for 10 min, and 40 cycles at 95°C for 15 s and 60°C for 1 min. These experiments were repeated three times with independent inoculation samples.

### Transient assay

The DNA sequence of Arabidopsis PR1 (AT2G14610) signal peptide was seamlessly fused in front of cDNA of the IGYP maturation protein (MbIGYP59, MbIGYP6, MbIGYP13, MbIGYP50, MbIGYP52, MbIGYP10, MbIGYP43, MbIGYP33, MbIGYP71, MbIGYP79, MbIGYP87 and MbIGYP94) by overlapping PCR, and then the PCR products were transferred into the binary vector PH35GY [44] using Gateway system (Life Technologies). All of the plant expression vectors were transformed into agrobacterium AGL1.

The transient expression experiments of *P. deltoids* NL895 and *P. tomentosa* were based on the Hybrid Aspen method [38] and modified slightly. In brief, the cuttings grew in MS medium for 4-6 weeks, then the cuttings, including root, were dipped in agrobacterium suspension liquid (Agrobacteria OD = 1.0, Acetosyringone 200 μM, silwet L-77 0.015%, and sucrose 2% in 0.5 MS), and infiltrated in continuous vacuum for 3 min (500 mmHg vacuum), then dried with filter paper and planted in MS medium with 50 μg/mL Cefotaxime. For Western blot analyses, total proteins (50 μg) were separated on 12% SDS-PAGE and subsequently transferred electrophoretically Hybond ECL membranes (Amersham). Membranes were incubated with a rabbit polyclonal anti-GFP antibody (Genscript) followed by an incubation with goat anti rabbit IgG conjugated with alkaline phosphatase. Immunoblot was visualized by use of BCIP/NBT substrate solution.

### Availability of supporting data

RT-PCR-seq data from this study have been submitted to GenBank under accession numberSRR1283188, SRR1283189 and SRR1283190.

## Electronic supplementary material

Additional file 1: **Summary of all MbIGYPs.** The table includes protein IDs, accession numbers of GenBank, conserved motifs, exon sizes and coverage of exon-exon junctions by RT-PCR-seq. (PDF 89 KB)

Additional file 2: **Figures that provide support information for the main text.** **Figure S1.** Schematic workflow of the RT-PCR-seq experiment. **Figure S2.** Alternative splicing discovered by aligning sequence reads with reference gDNA. The 10 panels show views with Tablet graphical viewer. **Figure S3.** Phylogenies of IGYPs, IGYAP1, ChiC and IGYAP2. **Figure S4.** Transient expression of β-glucuronidase (GUS) gene in *P. deltoides* NL895 and *P. tomentosa.* (PDF 1 MB)

Additional file 3: **Statistical data of** ***MbIGYPs*** **RT-PCR-seq results.** (PDF 60 KB)

Additional file 4: **IGYP, IGYAP1, ChiC and IGYAP2 homologues in Dikarya.** (PDF 143 KB)

Additional file 5: **Primers for RT-PCR-seq and real-time RT-PCR.** (PDF 65 KB)

## References

[1] Richards TA, Talbot NJ (2013). Horizontal gene transfer in osmotrophs: playing with public goods. Nat Rev Microbiol.

[2] Soanes DM, Richards TA, Talbot NJ (2007). Insights from sequencing fungal and oomycete genomes: what can we learn about plant disease and the evolution of pathogenicity?. Plant Cell.

[3] Perlin MH, Wösten HA, de Vries R, Ruiz-Herrera J, Reynaga-Peña CG, Snetselaar K, McCann M, Pérez-Martín J, Feldbrügge M, Basse CW, Steinberg G, Ibeas JI, Holloman W, Guzman P, Farman M, Stajich JE, Sentandreu R, González-Prieto JM, Kennell JC, Molina L, Kamper J, Kahmann R, Bolker M, Ma LJ, Brefort T, Saville BJ, Banuett F, Kronstad JW, Gold SE, Muller O (2006). Insights from the genome of the biotrophic fungal plant pathogen Ustilago maydis. Nature.

[4] Ohm R, Goodwin S, Grigoriev I, Consortium D (2013). Diverse lifestyles and strategies of plant pathogenesis encoded in the genomes of eighteen Dothideomycetes fungi. Phytopathology.

[5] Panstruga R, Dodds PN (2009). Terrific protein traffic: the mystery of effector protein delivery by filamentous plant pathogens. Science.

[6] Stergiopoulos I, de Wit PJ (2009). Fungal effector proteins. Annu Rev Phytopathol.

[7] Chiu R, Coutinho PM, Feau N, Field M, Frey P, Gelhaye E, Goldberg J, Grabherr MG, Kodira CD, Kohler A, Kües U, Lindquist EA, Lucas SM, Mago R, Mauceli E, Morin E, Murat C, Pangilinan JL, Park R, Pearson M, Duplessis S, Cuomo CA, Lin YC, Aerts A, Tisserant E, Veneault-Fourrey C, Joly DL, Hacquard S, Amselem J, Cantarel BL (2011). Obligate biotrophy features unraveled by the genomic analysis of rust fungi. Proc Natl Acad Sci U S A.

[8] Altmüller J, Alvarado-Balderrama L, Bauser CA, Becker C, Birren BW, Chen Z, Choi J, Crouch JA, Duvick JP, Farman MA, Gan P, Heiman D, Henrissat B, Howard RJ, Kabbage M, Koch C, Kracher B, Kubo Y, Law AD, Lebrun MH, O’Connell RJ, Thon MR, Hacquard S, Amyotte SG, Kleemann J, Torres MF, Damm U, Buiate EA, Epstein L, Alkan N (2012). Lifestyle transitions in plant pathogenic Colletotrichum fungi deciphered by genome and transcriptome analyses. Nat Genet.

[9] Salamov A, Shapiro HJ, Wuyts J, Blaudez D, Buée M, Brokstein P, Canbäck B, Cohen D, Courty PE, Coutinho PM, Delaruelle C, Detter JC, Deveau A, DiFazio S, Duplessis S, Fraissinet-Tachet L, Lucic E, Frey-Klett P, Fourrey C, Feussner I, Martin F, Aerts A, Ahren D, Brun A, Danchin EGJ, Duchaussoy F, Gibon J, Kohler A, Lindquist E, Pereda V (2008). The genome of Laccaria bicolor provides insights into mycorrhizal symbiosis. Nature.

[10] Ottmann C, Luberacki B, Kufner I, Koch W, Brunner F, Weyand M, Mattinen L, Pirhonen M, Anderluh G, Seitz HU, Nürnberger T, Oecking C (2009). A common toxin fold mediates microbial attack and plant defense. Proc Natl Acad Sci U S A.

[11] Gijzen M, Nurnberger T (2006). Nep1-like proteins from plant pathogens: recruitment and diversification of the NPP1 domain across taxa. Phytochemistry.

[12] Kombrink A, Thomma BPHJ: **LysM effectors: secreted proteins supporting fungal life.** *PLoS pathogens* 2013.,**9**(12). 10.1371/journal.ppat.1003769 PMC3861536 24348247

[13] Seidl-Seiboth V, Zach S, Frischmann A, Spadiut O, Dietzsch C, Herwig C, Ruth C, Rodler A, Jungbauer A, Kubicek CP (2013). Spore germination of Trichoderma atroviride is inhibited by its LysM protein TAL6. FEBS J.

[14] Chen H, Kovalchuk A, Kerio S, Asiegbu FO (2013). Distribution and bioinformatic analysis of the cerato-platanin protein family in Dikarya. Mycologia.

[15] Pazzagli L, Seidl-Seiboth V, Barsottini M, Vargas WA, Scala A, Mukherjee PK (2014). Cerato-platanins: elicitors and effectors. Plant Sci.

[16] Laugé R, Joosten MHAJ, Van den Ackerveken GFJM, Van den Broek HWJ, Wit APJGMD (1997). The in planta-produced extracellular proteins ECP1 and ECP2 of Cladosporium fulvum are virulence factors. Mol Plant Microbe Interact.

[17] Stergiopoulos I, Kourmpetis YA, Slot JC, Bakker FT, De Wit PJ, Rokas A (2012). In silico characterization and molecular evolutionary analysis of a novel superfamily of fungal effector proteins. Mol Biol Evol.

[18] Pedersen C, Ver Loren van Themaat E, McGuffin LJ, Abbott JC, Burgis TA, Barton G, Bindschedler LV, Lu X, Maekawa T, Wessling R, Cramer R, Thordal-Christensen H, Panstruga R, Spanu PD (2012). Structure and evolution of barley powdery mildew effector candidates. BMC Genomics.

[19] Liu H, Huang X, Pei G, Zhan G, Tang C, Cheng Y, Liu M, Zhang J, Zhao Z, Zhang S, Han Q, Han D, Zhang H, Zhao J, Gao X, Wang J, Ni P, Dong W, Yang L, Yang H, Zheng W, Huang L, Huang J, Wang X, Chen X, Zhao J, Guo J, Zhuang H, Qiu C, Liu J (2013). High genome heterozygosity and endemic genetic recombination in the wheat stripe rust fungus. Nat Commun.

[20] Spiers AG, Hopcroft DH (1983). Ultrastructural study of the pathogenesis of Marssonina species to poplars. Eur J Forest Pathol.

[21] Han ZM, Yin TM, Li CD, Huang MR, Wu RL (2000). Host effect on genetic variation *Marssonina brunnea* pathogenic to poplars. Theor Appl Genet.

[22] Zhu S, Cao YZ, Jiang C, Tan BY, Wang Z, Feng S, Zhang L, Su XH, Brejova B, Vinar T, Xu M, Wang MX, Zhang SG, Huang MR, Wu R, Zhou Y (2012). Sequencing the genome of Marssonina brunnea reveals fungus-poplar co-evolution. BMC Genomics.

[23] Cheng Q, Cao Y, Jiang C, Xu L, Wang M, Zhang S, Huang M (2010). Identifying secreted proteins of Marssonina brunnea by degenerate PCR. Proteomics.

[24] Fournier E, Gout L, Hahn M, Kohn L, Lapalu N, Plummer KM, Pradier JM, Quévillon E, Sharon A, Simon A, ten Have A, Tudzynski B, Tudzynski P, Wincker P, Andrew M, Anthouard V, Beever RE, Beffa R, Benoit I, Bouzid O, Amselem J, Cuomo CA, van Kan JA, Viaud M, Benito EP, Couloux A, Coutinho PM, de Vries RP, Dyer PS, Fillinger S (2011). Genomic analysis of the necrotrophic fungal pathogens Sclerotinia sclerotiorum and Botrytis cinerea. PLoS Genet.

[25] Jiang RH, Tripathy S, Govers F, Tyler BM (2008). RXLR effector reservoir in two Phytophthora species is dominated by a single rapidly evolving superfamily with more than 700 members. Proc Natl Acad Sci U S A.

[26] Jiang RH, Tyler BM (2012). Mechanisms and evolution of virulence in oomycetes. Annu Rev Phytopathol.

[27] Kale SD (2012). Oomycete and fungal effector entry, a microbial Trojan horse. New Phytol.

[28] Howald C, Tanzer A, Chrast J, Kokocinski F, Derrien T, Walters N, Gonzalez JM, Frankish A, Aken BL, Hourlier T, Vogel JH, White S, Searle S, Harrow J, Hubbard TJ, Guigó R, Reymond A (2012). Combining RT-PCR-seq and RNA-seq to catalog all genic elements encoded in the human genome. Genome Res.

[29] Grutzmann K, Szafranski K, Pohl M, Voigt K, Petzold A, Schuster S (2013). Fungal alternative splicing is associated with multicellular complexity and virulence: a genome-wide multi-species study. DNA Res.

[30] Gogarten JP, Townsend JP (2005). Horizontal gene transfer, genome innovation and evolution. Nat Rev Microbiol.

[31] Dewey CN (2011). Positional orthology: putting genomic evolutionary relationships into context. Briefings In Bioinformatics.

[32] Taylor JW, Berbee ML (2006). Dating divergences in the fungal tree of life: review and new analyses. Mycologia.

[33] Qutob D, Kamoun S, Gijzen M (2002). Expression of a Phytophthora sojae necrosis-inducing protein occurs during transition from biotrophy to necrotrophy. Plant J.

[34] Motteram J, Kufner I, Deller S, Brunner F, Hammond-Kosack KE, Nurnberger T, Rudd JJ (2009). Molecular characterization and functional analysis of MgNLP, the sole NPP1 domain-containing protein, from the fungal wheat leaf pathogen Mycosphaerella graminicola. Mol Plant Microbe Interact.

[35] Kleemann J, Rincon-Rivera LJ, Takahara H, Neumann U, Ver Loren van Themaat E, van der Does HC, Hacquard S, Stuber K, Will I, Schmalenbach W, Schmelzer E, O'Connell RJ (2012). Sequential delivery of host-induced virulence effectors by appressoria and intracellular hyphae of the phytopathogen Colletotrichum higginsianum. PLoS Pathog.

[36] Schouten A, van Baarlen P, van Kan JA (2008). Phytotoxic Nep1-like proteins from the necrotrophic fungus Botrytis cinerea associate with membranes and the nucleus of plant cells. New Phytol.

[37] Zhang B, Tong CF, Yin TM, Zhang XY, Zhuge QQ, Huang MR, Wang MX, Wu RL (2009). Detection of quantitative trait loci influencing growth trajectories of adventitious roots in Populus using functional mapping. Tree Genet Genomes.

[38] Takata N, Eriksson ME (2012). A simple and efficient transient transformation for hybrid aspen (Populus tremula x P. tremuloides). Plant Methods.

[39] Petersen TN, Brunak S, von Heijne G, Nielsen H (2011). SignalP 4.0: discriminating signal peptides from transmembrane regions. Nat Methods.

[40] Crooks GE, Hon G, Chandonia JM, Brenner SE (2004). WebLogo: a sequence logo generator. Genome Res.

[41] Hall BG (2013). Building phylogenetic trees from molecular data with MEGA. Mol Biol Evol.

[42] Trapnell C, Pachter L, Salzberg SL (2009). TopHat: discovering splice junctions with RNA-Seq. Bioinformatics.

[43] Milne I, Stephen G, Bayer M, Cock PJ, Pritchard L, Cardle L, Shaw PD, Marshall D (2012). Using Tablet for visual exploration of second-generation sequencing data. Brief Bioinform.

[44] Kubo M, Udagawa M, Nishikubo N, Horiguchi G, Yamaguchi M, Ito J, Mimura T, Fukuda H, Demura T (2005). Transcription switches for protoxylem and metaxylem vessel formation. Genes Dev.

