# Supplementary material for: Discovery of a novel small secreted protein family with conserved N-terminal IGY motif in Dikarya fungi
Source: BMC Genomics. 2014 Dec 20;15(1):1151. doi: 10.1186/1471-2164-15-1151 (PMC4367982; doi:10.1186/1471-2164-15-1151)
Supplement: Supplementary file 1 — Additional file 1: Summary of all MbIGYPs. The table includes protein IDs, accession numbers of GenBank, conserved motifs, exon sizes and coverage of exon-exon junctions by RT-PCR-seq. (PDF 89 KB) [file 12864_2014_6911_MOESM1_ESM.pdf]

Proten ID	Access no.[a]	Signal peptide	IGY motif	IP motif	RF motif	RxLR -like motif	Acid tail	First exon/second exon/third exon (bp)
MbIGYP1	XP_007295352	YES	KIVIGYAALTKDQA	VQMVVP	LRFS		YES	117/88/407
MbIGYP2	XP_007292647	YES	REVIGYRTVGEEEA	LQMVIP	LRFS		YES	120/103/446
MbIGYP3	XP_007288551	YES	REIIGYRTVSREEA	LQMVIP	LRFS	YES	YES	120/100/404
MbIGYP4[b]	XP_007292879	YES	RIVIGYRTVAEGEA	LQMLIP	LRFS		YES	114/103/452
MbIGYP5	XP_007294497	YES	RKIIGYRTVNKEEA	LQMVIP	LRFS	YES	YES	114/100/407
MbIGYP6	XP_007288958	YES	RKTIGYRSVSKEEA	LSMLIP	LRFS		YES	117/100/407
MbIGYP7	XP_007288835	YES	EVTLGYRTVSPEEA	LQMVIP	LRFS			108/97/443
MbIGYP8	XP_007288928	YES	TQVIGYAKLPGEKA	EQMTIP	LLFS			147/88/413
MbIGYP9	XP_007289173	YES	REIIGYAMIHESRA	YQMLIP	LRFS	YES		144/88/428
MbIGYP10	XP_007289300	YES	RILIGYRTVNEAEA	LQMAIP	LRFS	YES	YES	108/100/431
MbIGYP11	XP_007289897	YES	SQVIGYAMVSEEHA	LQMNIP	LRFS			123/85/428
MbIGYP12	XP_007290189	YES	RGIIGYRTVQEWEA	LQVVIP	LRFA	YES	YES	108/103/527
MbIGYP13	XP_007290348	YES	REVIGYRVVADSQA	LQMAIP	LRFL			117/100/458
MbIGYP14	XP_007290523	YES	RKIIGYQKLTRAKA	VAMLFP	VRFT	YES		108/82/434
MbIGYP15	XP_007290651	YES	REIIGWLTVAPAHA	LHMAIP	LRVS	YES		117/91/506
MbIGYP16	XP_007290690	YES	QIVIGYAKVPEDQA	MQMLIP	LRIS			150/85/389
MbIGYP17	XP_007290822	YES	MEIIGYRTASDAEA	LQMVIP	LRFS			108/100/425
MbIGYP18	XP_007290868	YES	RILIGYRLASPEEA	LQMVIP	IRFS	YES	YES	126/103/413
MbIGYP19	XP_007290932	YES	KMVIGYATVSKEEA	MQMLIP	LRFS		YES	117/106/434
MbIGYP20	XP_007290954	YES	KQVIGYAMVSKEHA	RQMTIP	LRFS			114/85/455
MbIGYP21	XP_007291014	YES	REIIGYRSVGDEEA	LQMVIP	LRFS	YES		108/103/407
MbIGYP22	XP_007291524	YES	KIVMGYATVPADVA	LRMLIP	FRFS			111/88/419

MbIGYP23	XP_007291663	YES	KEIIGYGTASQSEA	LRMVIP	LRFT	YES	YES	102/76/401
MbIGYP24[b]	XP_007291805	YES	QIIIGYAQLPEEQA	MQMLIP	LRFS			135/95/455
MbIGYP25	XP_007292311	YES	REVIGCRTVSDV	LKMDIA	LRFS			129/52/341
MbIGYP26	XP_007292553	YES	RRVIGYAIVSEDEA	MQMLIP	LRFS	YES	YES	129/85/431
MbIGYP27	XP_007293069	YES	RIVIGYRTVDPVEA	LQMLIP	LRFA			120/103/449
MbIGYP28	XP_007293167	YES	MKVIGYRTVNKWEA	VQMVIP	VRFA	YES		108/103/593
MbIGYP29[b]	XP_007293198	YES	KEVIGYRIVSPDEA	LQMVIP	LRFS		YES	90/100/428
MbIGYP30	XP_007293385	YES	IGQVVYDTVSPLEA	MQMVIP	LRFS			132/103/461
MbIGYP31	XP_007293414	YES	REVIGYRTVSQDEA	LQMVIP	LRFS		YES	105/103/590
MbIGYP32	XP_007293756	YES	RQIIGYRTVSAQEA	LQMVIP	LRFS	YES	YES	126/103/452
MbIGYP33	XP_007293967	YES	DQVIGYAKVSSRQA	YQMNIP	LRFS			117/91/476
MbIGYP34	XP_007293995	YES	RRNLGYGIVSQEEA	LQMVIP	LRLS	YES	YES	132/88/416
MbIGYP35	XP_007294003	YES	RETIGFRTVSQSEA	KQMFIP	LRFS		YES	120/103/416
MbIGYP36[b]	XP_007294053	YES	RVTIGYRTEEAE	QQMVIP	LRFS		YES	123/103/443
MbIGYP37	XP_007294177	YES	RQIIGYRTVSKLEA	MQMLIP	LRFS	YES		153/100/413
MbIGYP38	XP_007294299	YES	RELLGYRIVPQEEA	MTMAIP	LRFS	YES	YES	105/97/407
MbIGYP39	XP_007294346	YES	EELIGYRAASEEEA	LEMLLP	LAYT			105/97/443
MbIGYP40	XP_007294402	YES	DSVIGYATVSAEAA	LRMLIP	LRFS			111/82/419
MbIGYP41	XP_007294405	YES	QIVIGYARVPEDQA	MQMLIP	LRIS			150/85/386
MbIGYP42	XP_007294406	YES	GEVIGYASVLEDEA	LQMLIP	LRFG			132/88/410
MbIGYP43	XP_007294442	YES	RELIGYRAVTEDQA	PQMVIP	LRFS	YES	YES	120/103/470
MbIGYP44	XP_007294631	YES	RKIIGYRTVDEEEA	MQMVIP	LRFS	YES	YES	105/103/425
MbIGYP45	XP_007294919	YES	REIIGYRTVTAEEA	LIMVIP	LRFS	YES	YES	129/103/449
MbIGYP46	XP_007295237	YES	REVIGYRTVSRKEG	WQMVIP	LRFA			105/97/446

MbIGYP47	XP_007295262	YES	AIVIGYATLTEEEG	LLMVVP	LRFS			102/88/434
MbIGYP48	XP_007295536	YES	REVIAYRTVSEEEA	LQMVIP	LRFS		YES	108/100/1058
MbIGYP49	XP_007296451	YES	RKTIAYRTVSQAEA	LQMVIP	LRFS		YES	117/103/440
MbIGYP50	XP_007296492	YES	MTTIGYRTVSRAEA	LHMLIP	LRFS			114/97/515
MbIGYP51	XP_007296640	YES	RRIIGYRTVSRAEA	LQMIIP	LRFL	YES		111/103/413
MbIGYP52	XP_007296670	YES	RKIIAYRTVSPMEA	IQMVIP	LRFA	YES		102/103/548
MbIGYP53[b]	XP_007296693	YES	RKVIGYRTVAKVHRDEA	LQMLIP	LRLS	YES	YES	111/96/410
MbIGYP54	XP_007296706	YES	KKVIAYRTVAKEEA	LQMVIP	LRFS	YES	YES	102/109/461
MbIGYP55	XP_007296782	YES	REIIGYRIAHEDEA	LQMLIP	LRFS	YES	YES	114/100/473
MbIGYP56	XP_007297128	YES	QEIIGYRTVFREEA	QQMVIP	LRFS			120/103/434
MbIGYP57	XP_007297319	YES	HVLIGFTVLSEDQA	WQMLIP	VRFA	YES	YES	117/139/536
MbIGYP58	XP_007297356	YES	RKTIGYRIVSKGEA	LHMLIP	LRFS	YES	YES	120/103/467
MbIGYP59	XP_007297373	YES	RRLLGYRTVSEDEA	MQMVIP	LRFS	YES	YES	108/103/506
MbIGYP60	XP_007297374	YES	REFIGYRVVYDREA	FQMVLP	IRFS	YES		126/97/467
MbIGYP61	XP_007297403	YES	RIVIGYRTVSEAEA	IQMLIP	LRFS		YES	102/103/416
MbIGYP62	XP_007297412	YES	REIIAYRTVSRAEA	LQMVIP	WRLA	YES		105/103/536
MbIGYP63	XP_007297703	YES	REVIGYRTVSPWET	HQMVIP	FRFA			105/103/627
MbIGYP64[b]	XP_007292967	YES	KIVIGYAALSEDEA	LQMVIP	LRFS			117/88/443
MbIGYP65[b]	XP_007295406	YES	REIIGYRTLSSEA	VQMLIP	LRFS	YES	YES	114/97/566
MbIGYP66[b]	XP_007297022	YES	QTVIGYTTVSESQA	LQMAIP	LRLS			126/88/488
MbIGYP67[b]	XP_007297431	YES	RELIGYLLATGGEA	IQMLIP	LRFS	YES	YES	105/97/504
MbIGYP68[b]	XP_007292248	YES	RELLGYITVTPEEA	MQMLIP	IRFS			108/88/455
MbIGYP69	XP_007290578	YES	N/A	LQMLIP	LRFA			66/103/452
MbIGYP70	XP_007293242	YES	IEVIGYRTVSSTEA	WQMVIP	VRFS		YES	135/94/437

MbIGYP71	XP_007295772	No	RQIIGYRMVRPEEA	LQMVIP	IRFA	YES	YES		153/103/452
MbIGYP72	XP_007294419	YES	GIVIAYRTVSPAEA	LQMLLP	LRFS				99/103/389
MbIGYP73	XP_007290873	YES	MKIIGYATAKPEQA	KQMLIP	LRFS	YES	YES		120/91/425
MbIGYP74	XP_007290873	YES	QMVVGYASVPRELA	LQMLIP	FRFA				129/82/410
MbIGYP75	XP_007289443	YES	MKVIGYARVPPGTA	MQMLIP	LRFT	YES			114/88/377
MbIGYP76	XP_007289555	YES	REVIGYAMIPRSEA	PIVLIP	LRFA		YES		111/97/401
MbIGYP77[b]	XP_007294094	YES	RQLIGYAIASEEQA	LQMLIP	LRFS	YES	YES		123/100/452
MbIGYP78	XP_007294892	YES	KQVIGYAMVSEEHA	RQMTIP	LRFS		YES		120/85/446
MbIGYP79	XP_007289774	YES	DRVIGYAMVTGRQA	HHMNIP	LRFS	YES	YES		117/85/428
MbIGYP80	XP_007295515	YES	KRAIGYATIPEEVA	MQMLIP	LRFS	YES	YES		123/88/434
MbIGYP81	XP_007296701	YES	PITIGYATVTPDVA	LQMLIP	LRFS				123/91/407
MbIGYP82	XP_007288763	YES	SKVMGYATVSEADA	LQMLIP	LRIS	YES	YES		123/94/383
MbIGYP83	XP_007290672	YES	GKAIGYASVSAELG	MQMLIP	LRFS	YES	YES		126/91/434
MbIGYP84	XP_007293724	YES	RRIIGYATVSRREA	SQMVIP	LRFS	YES			147/88/410
MbIGYP85	XP_007290819	YES	PSVIGYAKLPAAHA	LLMLIP	N/A				138/94/506
MbIGYP86	XP_007289824	YES	NELIGYAKVSPGLA	AQMTIP	LRFS				117/88/527
MbIGYP87	XP_007294861	YES	DTVIGYALVSEEDA	LLMSVP	LRFM				129/88/416
MbIGYP88	XP_007292534	YES	RQVIGYTTVPEEMA	LQMAIP	LRFS		YES		123/88/446
MbIGYP89	XP_007295016	YES	NQVIGYATVSEEQA	LQMLIP	LRFS				129/106/428
MbIGYP90	XP_007288348	YES	RKFIGYARVSESLA	LQMAIP	LRFS	YES			111/82/473
MbIGYP91	XP_007289856	YES	HTVIGYATVPSEEA	LHMLIP	LRVS		YES		123/88/515
MbIGYP92	XP_007293694	YES	RIVIGYAIVSEEQA	LQMAIP	LRFA				117/88/425
MbIGYP93	XP_007289174	YES	KIVIGYAVVIKEYA	MQMVIP	LRFS				117/91/413
MbIGYP94	XP_007294286	YES	EIVIGYTAVTSDQA	LQMVIP	LRFS				117/88/497

MbIGYP95	XP_007289546	YES	RIIIGYRTVSEEEA	LQMVIP	LRFS	YES	YES	114/112/425
MbIGYP96	XP_007289562	YES	RVVMAYRTASQAEA	LVMNIP	LRFS			108/97/413
MbIGYP97[b]	XP_007296908	YES	REAIGYVTVSHGAA	LQMVIP	LRFS		YES	129/76/434
MbIGYP98[b]	XP_007289201	YES	RQVIGYRTVDKDEA	LQMLIP	IRFG		YES	105/103/436
MbIGYP99	XP_007293725	YES	RGLIAYRIVSEDVA	LQIVIP	LRFS	YES	YES	105/94/389
MbIGYP100	XP_007288304	YES	DVLIGYRTVSEAEA	MEMLIP	IRFS			105/100/425
MbIGYP101	XP_007289953	YES	MMVIGYAAVSRWEA	LQMVIP	LRFS			144/88/434
MbIGYP102	XP_007289544	YES	SEIMGYRTVSKEEA	LQMVIP	LRFS			102/103/422
MbIGYP103	XP_007288081	YES	KILIGYGVVSSKEA	LQMNIP	LRFS	YES		117/88/491
MbIGYP104	XP_007292925	YES	REVIGYRTASKEEA	TQMLIP	LRFS		YES	120/100/422
MbIGYP105	XP_007289456	Yes	RMLIGYRTVNEEEA	LQMVIP	LRFS			111/103/431
MbIGYP106	XP_007291574	No	N/A	LQMLIP	LRFS			103/50/447
MbIGYP107	XP_007295236	YES	REIIGYRTVTAEEA	LIMVIP	LRFS	YES	YES	114/103/420

[a] Accession numbers of GenBank

[b] Adjusted based on RT-PCR-seq results. Adjusted 13 gene models as following:

Exons are shown by red fonts and introns are shown by black fonts.

>MbIGYP4

ATGCCACTCCCGAGATCGATTTGCGCGATCCTGCTAGCCTTAAGCTTTCTCTTTGATGGAGCGCGCGGAGTAACTCCAGGACGGATAGTGATCGGCTACCG
AACCGTTGCCGAGGTAGGTTGGTTTTGGTTTCACTTGGGTTCACACAGGATAAATAATATAAGCTTATACCGACTTACGCGAATCATCTATCATAGGGGGAAG
CCGAGGATATCAACGAGGATAACAAGCCGTATAGGGAAGCACTGATCGATGAAGCTCGCCAGAAGCTTAATCAGCTGGGAAACGGCCTACATATCAGTAAAT
CAATTTCATATTTTGTACCACTGCATGTGTCACGCTCTTGTTGCGCTCAAGCTGATAGACTCTCAATAGTGCGGGAGCCGGTTAGCTGGGAGGGCTTTCCGT
GGTGGCGCAACTGGTACTGCGTCATCGAGGCGGATATCAACAAGATTGCCAAAGCGCACAAGATATACATCCCCGAATACTACCAACAGACGGACTGGGA
CGGCCAGCGTAGCGATGTGGTACTGTGGCACGGAGGCGAAGACGTTATCGTGGATTACATCACGCAGGGTGTGCCGAAAGACCCCGGGAAGGCGCTGC

GCTTCTCGTGGATCTCGGATTTCGACTGGGAGCTGCAGATGCTCATCCCCACCGCGGTGGTAAACAACGACGAGTTGGACCTGTGGTCTGAATGCTTCGA
GACGAAGGACGAGCTGAAGGAATACTCGAGCGAGGTTGTCGATTGGGAGAGCTGGGGTATCGCGGGGGATTGTGGACTACCGAGTCTGGAAACCTTCGT
TCCGTGGATAGACGATGTTTGA

>MbIGYP24

ATGCTACGGTCAAAGTCTATCTTCACGATCTTGCTAACCTTGAGCTTTTTATCTGAAAAGGCTAAAGCTCAAGGATTTTGGTGGTGGCGCCAACCAACAGA
ACAGATAATCATCGGCTATGCACAACTTCCCGAAGTAAGTTTCACTTTGATTTACCACTCCTCCTCCCGAATAAATCCCGCCTGGGGGTGGCTCAAAAGCT
TAAAACTCTACCTTAAACCTGGTTTCCCTACCCTCAGGTGGTAATTACTACCACTATTACGCCCGTGAGGTTGTATATAGCATAAGCTTATATTAATCGCGC
AGATTATAGGAGCAGGCTAAGCGCATCAATCAAAGCAACAAGCTCTATGTAGACGAATCCCCATACCTTAATCGATTAGGACTTGGCTTTTATCTAAGTGA
GCTGATCTATATACTTTGGATCGTTATCCATTACGCGAAGCTAATAATCCAAATAGTAAACGACCCTAGGGTCTGGCTAGGTAAAGAAGGGGGTTGGTATT
GTGCTATCAAAGCGAATGAGAGTAAGATGAGAAATATTGAAAAAGTCTATATCCCGAAATCTTATGAACAAAAGATTTGGAATCGTGTTGAGCAAGTAAATT
TATGGGGCGGAACCGAAGAAGTCATTGTGGATTACATTGAATTCGAGTCAAAGCCAAAAATATTACAGCCCGAAAAAGCGCTGCGCTTCTCCCTCAATTG
GCAGATGCAAATGCTTATTCCAACGAAAGTGGTAGATAACGGTGATTTGGATTTATGGGGTAAATGCTTTCGATCTGAGAATGAGCTGAAGCAATTTTCGT
ATAATATTATTGATTGGGAATCTTGGAATATTAAAGGAGATCGTGGAAAACCGGTCTATGATCCTGTTTTTAGGTTCGGAAAAGGGGGGGAGGTCACTTGG
TGGTAG

>MbIGYP29

ATGCTCCTTTACTCGATCTTGCTAGCCTCGAGCGTCCTCTTCAACACAGCTTATGGAAAAGAGGTTATCGGTTATCGAATTGTTTCTCCTGTAAGTTTCACTT
GATTTATATATTATAAGCTTATATTAATTACGCGAATTATAGGACGAAGCCGAGGGGATCAATGATAACAATAAGCCTTTTGACTATAAAGCATACGATGATGACT
CATTGTCTCCTCAATTGGGACGCGGATTTTATATGAGTAAGTTGATTTATGTTTATCATAAATCGCTACTCATTATGCGGCGTTAACGACTCAAATAGCAAACGT
ACCTGATAGCCAGCTGGCTCTTTCTGACGAATGGTATTGTGTTATCAAAGCGGATAGTGATAAAATTGGAAAGGCAGACAAAGTATGGATCCCGAAAATCTTT
GAGGATGAGGAAAGGTCAGTGGAATTATGGACCGGAAACGAAGACGCTATCCTCGAGTATATCAAGTCCCTGGTGTCGGATGACGCGGAAGAGGCATTGC
GCTTTTCATACATAGTAGGCGCTGGGGAGAAGCTGCAAATGGTTATTCCACCTGAAGCGATAGACGAGGATGACTTGGGTTTATGGGCGCAATGCTGGGCA
ACAGAGGAGGAGCAAATGAGACATTCGAGCGAAATCGTTCCATGGACGACTTGGAATATTGTTGGAAATCTTGGGCCGAGCGATCCAGATGAAATGGAAAC
GGAAACGGGCTGGTGA

>MbIGYP36

ATGCCGCCGCTAGCGAAGTCCATTTGCGCAATCTTGCTAGCCGTGAGCTTTCTCTTTGATAGAGCTCGAGGATTTCCCTCAGGAGGAACTAGGGTAACCA
TCGGCTATCGAACAGTAAGCCGAGTAAGTTTCACCCGATTGCATCGTCGGTCTACCTTTTTGCGCTCGATCGGTACTCTTTTCTTCTTTACAATAATACTTA

AAAAGGCAACCGGTGATCACAGAGCATGCATAAATACTAATCACGCGAGTTGTAGGAAGAAGCCGAGCTTATCAATTTTCTCCGTCAGCCATTTAGAGATA
GAGAATATGATGAAGAAGCTGATGGCTTGAATCAATTAGGACATGGCTTTTACATGAGTGAGTTAAACTTCGACTTCTGGATCGCTCGTTCTTTTATCAACG
CGACAGCTAAGACTCCCGTTAGTAAACGAACCTGCTGGCTGGCCAGGTCGTCCATTGGAGGATAATTGGTTTTGTGTTATCAAGGCAGATGGTGATAAGG
TTAAGAATGCGGCCAAAATTTACATCCCAGAACGTTATTCGAAATTGACCTGGAATGCTCGGACTGAGGAGGTATACTTGTGGGCCGGAGAAGAAGAAAC
CGTCCTTGAATACATCGAATCGATGATCCCAAACCCCGAGCGGGCGCTGCGCTTCTCATGGATCGTAGCTGGAGGTTCGATGCAGCAAATGGTCATTCC
AAGTGATATAATAAATTACAATGAGCTGGATATGTGGGCCCAATGCTTTCAGACAGCGGAAGAGCTTTATCGATGTTCGAGCGAAATCATTGATTGGAAGA
GTTGGGAGATTACTGGGAATCCTGGAATACCTGGCTCTGCAAATTACATTCAACCGGATTTCTGA
>MbIGYP53
ATGCTACTATCGACGTCTATCTGCGCGACCTTGCTAGCCTTGAGCTTTTGCTTTAGTAAAGCTGAAGCAGACGATAACAGGAAAGTCATCGGCTACCGAACA
GTTGCCAAAGTAAGTTTGTTTTACCTGATTCATATATAGGATAAGCCTATACTAATTCCGCAAATTATGGGCGGAAGCCGATCTCATCAATGAAAAAAACGAGG
TACATAGAGATGAAGCCTTCGATAACGAAGATTTTTACCTTAACTCAATAGGATTTGGCGTTTATATGGGTGAGTTGATTTATACAGATCTTTGCATATTTCGCG
GAGCTAATAATTCAAATAGTACAAGAACCTGCCGGCTGGTTTGGCCAAGAGTCGGATTGGTATTGTGTTATTAGAGCGGATATTGAGAAGATAGACAGTATAG
ATAAAGCGTGGATCCCATTGACTTGGGAAAAAGAAAACGAAGACGGAAAAGTTGAGAAGATTGAATTATACGGTGAAGAAGAGCTCACCGTGGAATACATC
AGGTCATTGATGATACCAGAGCCGGAGAAAGCGTTGCGTTTATCAACGATCGATGACGAAGGGAGTCTGCAAATGCTTATTCCAACGAAAGTAGTAAATAGC
GGTGTATTGGATTTATGGGCCATCTGCTTTGAAACGCCAGAAAAACTGGCAGAATTTTCGAACGAATTCCCTGAATGGGACGATTGGGATATCAAAGGAGAA
TCTGAAGCTGCTTTTATGTGA
>MbIGYP64
ATGCTACGATCGAAGTCTATTTTCGCGATCTTGCTAGCCTCGAGCCTTCTCTTTAGTAGAGCTCAAGGATTTTGGTGGCGCCAAAAAATAGTCATCGGCTATG
CAGCACTTTCCGAAGTAAGTTTCACTCGATTAATGTAGCATAAGCTTATACTAATTACGCGAATTATAGGATGAGGCTACGCGGATCAATGAAGACAACAAGCT
ACATGTACCTGATGAGTCCTCACAGGATCAATTAGGAAGTGGCTTTTATATGGGTGAGTAGATTTAATAGATCGTTACTTATTACGCGGAGCTAATAATCTGAAT
AGTCAACGAACCTGGTAACTGGCCGAGTGAAGCAGGGCTTTGGTATTGTGCTATCAAAGCAAGACGAAGGAGGATAAAACGTGCTAGCAAAGTCTACGTC
CCGAGGTCGTATGAAAAACTGACCTCGAATGGTGTTGAGCAACAACCCTTATGGTTCCAAGACGAAAAAATCATCGGGGAGTACATCCAGTCACATGCATT
GATGTCAGATCCCGAGAAAGCACTGCGTTTCTCACGAATTATGAATCTCAAGAAGCGGCAGCTGCAAATGGTTATTCCAACGAAAGCGGTAGAGGATAATAA
GTTGGATTTATGGGCCAAATGCTTTGAATCAGAAGAAAAGCTGAAGAGGTTTTCGAAGGGTGTAATTGATTGGGAAGGTTGGACGATTAGAGGAGAGAGAG
ATAGGTCACAAACGGGCTTGCCTAATGGGGTGGTAAGGTAA
>MbIGYP65

ATGATCCTATTATCGCAGTCTATTAGCTCGATCTTGCTAGCCTCAAGCTTTCTCTTCACTAGAGCTTGCGGAGAAAGAGAAAGAGAAATTATCGGTTATCGAA
CCCTTTCCTCAGTAAGATTCACTTGGTTTATATAATATAAGCAAATATTAATCACGCGAGTCATAAAAGGAAGCCGATAATAGCAATGATTATGAAAGGCGTTATA
GAAATGCAGAATATGATGAGGAAGCTACAACTGCTCAAATAGGAGCTGGGTTTTATACGTGTAAGTGGATCTATTTCTATCGATCATTACTTATTACGCGGAGT
TAATAATCTAAATAGTCAACGAACCTGCTGGCTGGCAGGCGAGAGGGAACGAATGGTATTGTGTCCTCGAAGCGGATAGTGAATTGCTTAAAAAGATAAGCA
AAGTATGGATCCCAAAATCCTACACGAAAAGCACCGGGGATGGTGAGACACAATTATGGCACCGAGACGAAGAAACTCTCTTGGAATATATCCAGTCAATAG
TGCCAGAGTCAGACCCGAAGAAGGCATTACGCTTTTCAGCAATTCCATCTGTCCCTGGGAAGGTGCAAATGCTCATTCCAAGTGAGACGATCGAAGATGAG
TTAGAAATATGGTCTCAATGCTTCGAAACAGAGAAGGAGCTAAGAAGTAATGTGAACGTAACCGTTGATTGGATGAGTTGGGGTATTGCTGGACCGAGACC
GAGCGGTGTCTCTACGAAGGCAAAGGGAGGGAGCTGGTATCGGCGCTGGTTAGATTGGTGGCATTCGTGGCGTTCGTCGGGTTCGTGGCGTTCGTGGC
GTTCGTGGCGTTCGTGGTTTTCGTGGCGCCCGTGTTGTCGAACGGAATCGCCCCCGAGGGAGCATCCGCTTCGGTGAAGATGGACCATCATCAGCACCA
GCACCAGCACCGCTGTAAGAAATTCCGTAGTGGTTGA

>MbIGYP66

ATGCTACGATTGAAGTCTATATTCGCGATCTTGCTAGCCTTAAGCTCCCTCTTGGGTAGAGCCCAAGCATTTTGGTGGGAAGAGTGGCTCCAACAAACAGTC
ATCGGCTATACAACAGTTTCCGAAGTAAGTTTCACCTGATTTATATAGCATAAGCTTATACTAATTACGCGAACTAAAGTCCCAAGCTCTGCGCATCAATGAAC
ATAATAAGCCAGTTGTACAAGAATCCGAATACCGTACCCAATTAGGACCTGGCTTGTACATGGGTGGGATCATTTATAGAGATCGTTATCTATTACGCGGAGCT
AATAATCTAAATAGTAAACAGAGTTGGTACCTGGAAGGGTGATGAAGGGAGTTGGTATTGTGCTATTAAAGCGAGAAATTGGAAGATGAAAATGATCGGCAAA
GCTTTCGTCCCTAAAACTTTTATGGGAACTACCGTCAATGGTATTGAGCAAACCCAATTATGGGGCGCAGACGAAGAAGTCATCGTGGAATACATTCGGAAC
AAGCTAGGGATGTCACACCCGGCGAACGCGCTACGACTTTCCTGGGTTTTGGGTATAAAGTGGCAGCTGCAAATGGCTATTCCAAAGAAAGTGGTAGATGA
GGATAAATTGGACTTATGGGCCCAATGCTTTAGAACAGAAGCTGAGCTGTACGCTTTTTCGAACAAAGTTATTGATTTTGAGGCTTGGACCATTGCAGGAGA
TCCTGGATGGCCGACCTCCATTCTTCCAGAAAGACCTAGACAAGCCGACGACGACCTACAATCAGAAGGGAGTGGCTCATCAGTTGTATATTAG

>MbIGYP67

ATGAGGCTGTCGTCGCAGTTAATTTGCTCGATTTTCCTAGCCTCTAGCTCTCTCTTTAAGGAAGCTTATGGAAGAGAACTTATCGGTTATCTACTTGCTACTG
GAGTAAGTTTCAATTGATTTATATAATTTAAGCTTATGTTAATTACGCGAATCACAGGGGGAAGCCGAGTATATCAATGGTGAAGAAAAGCCTCCTAAAGATGA
GAATTTCTATAAAGGATTTATGAAAAAACTAGGATTAGGCATTCACATCGGTGAGTTGGATCAAATGTATAAATCGTTATTCATTACACGGAGCTAATAATTTGAA
CAGTCAATAAACCTGCTACCTGGTGGGACGGCAGAAGAGCATATTATTGCGCTTTCGAAGCGGATCTTGAAAAGATGAAAAAATTGGGCAGAGTATGGATC
CCACCTAAAAACTATCAGAGTGGTCAGTCATTATGGTACGCCAACGAAAAAACTATCATGGATTATATCAAGTCAGTGGTGTCAGATGACGCGGATGAGGCAT
TGCGCTTTTCATACAGTGGTATTTCGGATGACCCTGAGGACATTCAAATGCTTATTCCAATTAAATCGCTCAATAAAGATGACTTGGATCTGTGGGGTCATTG

CTGGGGAACAAGGGCCGAGCTTGATCAACAGTATGAGAGCCAAACCGTCAATTGGGAGAGTTGGGGGGATTTTGCTGGAGATCCTGGACCGAGACCAAG
AAAGGCAAGGCCGGATAGAACCTGGGATCGGGTGGAGTAATGGAAGAGCTTAAAGGAAGGAAGTACCCAAAGAAGGGAGAAAGGAATGCAGGGAACAGT
AG

>MbIGYP68

ATGATTCTATTACCGAAATCCTTTTTCGCAATTTTGCTAGCCTCGAGCTTTCTCTTTAATAGAGCTCAAGCAATAAGAGAACTTCTCGGCTATATAACCGTTA
CCCCAGTAAGTTTTACTTGATTTATATAATATGAGCCTATACTAATTACGCGGATTATAGGAAGAAGCCATGAATATCAATTCTTTCCGCACTCTATCTGGAT
TCGGTCTGCAAAAACAAGGTATGATAGGAGAAGGTATTTACTTTAGTAAGTTAATTTATATTTTTAAATCTTCACTTATTACGCTAAGCTAATAATATATGTAG
GACTAGAAATTGAAGCTTGGGGTTGGGACGAGGTGGACCCAGATGGTATTGTGTTGCCGATGCCCATAGGGGTAAGATGGACAAGGTGGAAAAAGTCT
ATATCCCAGAACTTTGGCCAAAGCCGATTGAGAATGGTGTGTATTTGATGACCGCATTATGGAACCGAGGCGAAGACGCTATCAGGGAGTTTATCCTCAA
TTTATTTGATAGAAGGAAAAACCCCGACAAAGTAATACGCTTCTCAAGGATCCCAAATTTCGGTTGGGATATGCAGATGCTCATTCCAACTTATTACCTGCA
AAATGAAGAGTTGGGTATAAAGAGTACATGCTTTGAAACAAGGGAAGCCCTAAAAGATTATTCGAACAAGCCTGCTGATTGGAGCAGATGGATTAGTACTG
AAAATCCTGGGGACTTGGACATTAATACTCAGGATCTACCCGAGCATCCTTGA

>MbIGYP77

ATGCCACAGTCGAACTCTATTTTCGCCCTCTTGCTAGCTTTAAGCTTGCTCTTTAGCCGAGCTCAAGGGGGTTTTTGGCGTAGCGATGGACGCCAACTCAT
CGGCTATGCAATAGCTAGCGAAGTAAGCTTCACTTGGTTTTACACGGAGTACTATCGCACAAGGAAGCTTCTATTAATCAGGCGGAACTATAGGAGCAGG
CGAGGCTTATCAATGATGGCCATAAGCCACGACTCCTGCCACCAGTCGAGGGATCCGAACCCGCTGGTGCATTAGGAGCGGGCTTTTATATGCGTGAGT
TGATCTACCTACAAGTATAGATCGGTCCCTATTACGACGCGAAGCTAAGGAGTCTAAATAGTGAACAAGCCCTCTGGCTGGAGAGCTACAGAAGAGAGTT
GGTATTGTGCTATCAGAGCGAAGAAGAGTAAGATAAAGAAGATCAGCAAAGTCTGGATCCCGAAAGCATATGAAAAGAGAACTGAGGAGGGTATTCTGGA
TCAGGAATTATGGGACCAGAGCGAAGACGTCATTACAGAGTACATCCAAGCAGAGGCATCGATAGATGATCCCGAGGCTGCGGAGAAAGCGCTGCGCTT
CTCATGGGTTATGGGTGCCAGCGACTGGCAGCTGCAGATGCTCATTCCAACGAATGTGCTAAATAACTATAACCTGGATTTATGGGCCAGGTGCTTTGAC
TCAGAAGATGAACTGAAGAAACGTTGGAACTATGTCGTTCCATGGGACAGCAGCAGTAGATGGGATATTGAGGGAATTCGTGGAAATCCGAACTGGTGGT
TTGTTGCGGAGCACACTGAACGAGTGCCCGCTGCATCACGCTGA

>MbIGYP97

ATGCTACGACCGAAGTCTATCTTCTCGATCCTGCTAGCCTTAAGCGTTCTCTTGAGTAAAGCTCAAGGGAGGTGGCACTTTGGGCAATTGCATGCAAGAGA
AGCCATCGGCTATGTAACAGTTTCCCATGCAAGTTTCACCTGATCTATATAATATACTTAGGCTTATATTAATTACGCGAATTATAGGGCGCAGCTCTTAGAATC
AATGAAAACAATAAGCTCTTTTCTGACGAAACTTCATTAGGGTCCGGCTTTAATATGAGTGAGTCTGATCTATATAGGTCGTTACTTATTACGCGGAGCTAATC

ATCTAAATAGTAAATGAGCCTGGTGTCGGTGCAGGAGGTTGGTATTGTGTCGTCGAAGCGAAACCGAAGAAGATTGACAAAATCGATAAAATCTACATCCCG
GAATCTTATGACCCGTGGACCCCGCGGGGTAGAGTGCGACGACAGTTATGGGACGGATCGGGAGAATTCGTCTTGGATTACATCAGGTCAAAACCGTTGA
TATCAGACCCCGATAAAGCGCTGCGCTTTTCATGGTTTCAGTATGAGGGTGCCGAATGGCGCCTGCAAATGGTTATTCCAGCGAGAACGATATCAGAAGGT
AATTTGGGTCTATGGGCTAAATGCTTTGAAGCACAACGTGATCTGATGGCATATTCGGACAAAGTTATTCAATGGGATACTTGGACCATCGAAGGAGAACTAG
GACAGCGACCGGACGCGGATCGCAGGACCTCTTCCTAA
>MbIGYP98
ATGCTACTATTATCGAAGTCCACTTGCGCAGTCCTGCTGGCCTCTAGCTCTCTTTTGAATAGAGCTGAAGGAAGACAAGTTATCGGCTATCGAACAGTTGAC
AAAGTAAGCTTCACTTGATCTAATCGTCCGTTACCGACAATTACATACGATGCCTAGCATAAGCTTACGCTAATTACGGGGATCATAGGACGAAGCCGACGTG
ATTAATACTTTCGGAAAGCCGTTCAGAGATAAAATATTCGACATGGATGATACTTTCCTTCACCAATTAGGAAATGGCTTTTACATGAGTAAGAAGTTTGATATT
TATATCTATATATCGTCCCGTCACTTCTATACGCGAAGCTAATTACTTTCAAATAGCGAACATGCCTGCTAGCTGGGAGGGTGATCCATTGGATTGGTATTGTG
TCATCGAAGCGGATACTCACAAGATTATAGAGGCGCCCAAAATATTCATCCCAGAATTTTGGGACATACCGACTGAGGCTGGGCCTGAACGAATGTACCTAT
GGTCCGGAGTGGAAGGACTCATCTTGGAATACATCGGCTCGAAAATCTCCCACCCTAAGGAGGCGATTCGCTTTGGGTGGATCTTATTTAGGGAATGGGAG
TTGCAAATGCTCATTCCAACTGATTTGTTGAACGATGATAAGTTTGATTTATGGGGTGAATGCTTCGAAACAAAGGATGAACTAAGGCAACGTTCGAACGAAA
ATATTGAATGGACGGCTTGGACTATTAGTGGAAATCCTGGATACCCGAGCGACGAAGGTGTTTTGTAGTAAGTCGGGGAGTATGA
