# Supplementary material for: Discovery of a novel small secreted protein family with conserved N-terminal IGY motif in Dikarya fungi
Source: BMC Genomics. 2014 Dec 20;15(1):1151. doi: 10.1186/1471-2164-15-1151 (PMC4367982; doi:10.1186/1471-2164-15-1151)
Supplement: Supplementary file 2 — Additional file 2: Figures that provide support information for the main text. Figure S1. Schematic workflow of the RT-PCR-seq experiment. Figure S2. Alternative splicing discovered by aligning sequence reads with reference gDNA. The 10 panels show views with Tablet graphical viewer. Figure S3. Phylogenies of IGYPs, IGYAP1, ChiC and IGYAP2. Figure S4. Transient expression of β-glucuronidase (GUS) gene in P. deltoides NL895 and P. tomentosa. (PDF 1 MB) [file 12864_2014_6911_MOESM2_ESM.pdf]

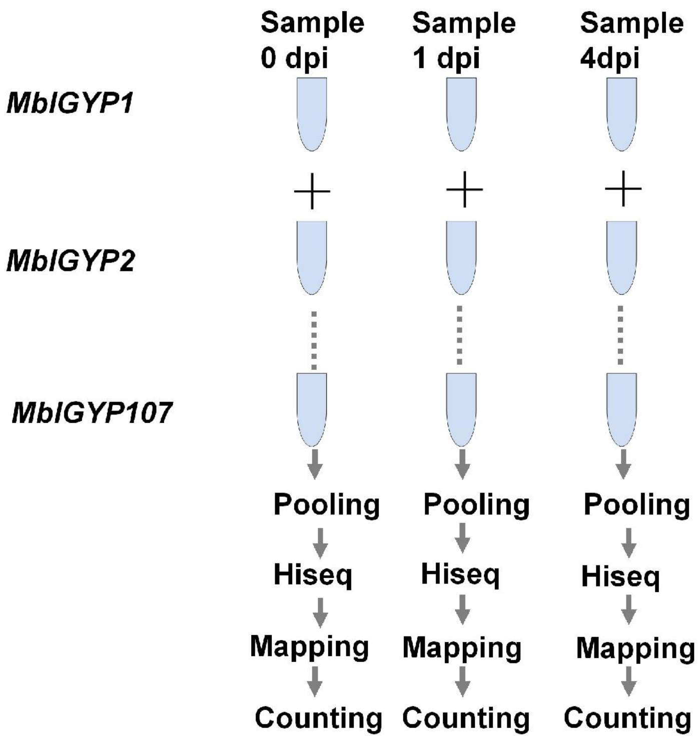

Figure S1 Schematic workflow of the RT-PCR-seq experiment

The cDNA fragments of 107 *MbIGYPs* were amplified with 0 dpi, 1dpi and 4 dpi samples. PCR reactions were shown as light blue cones.

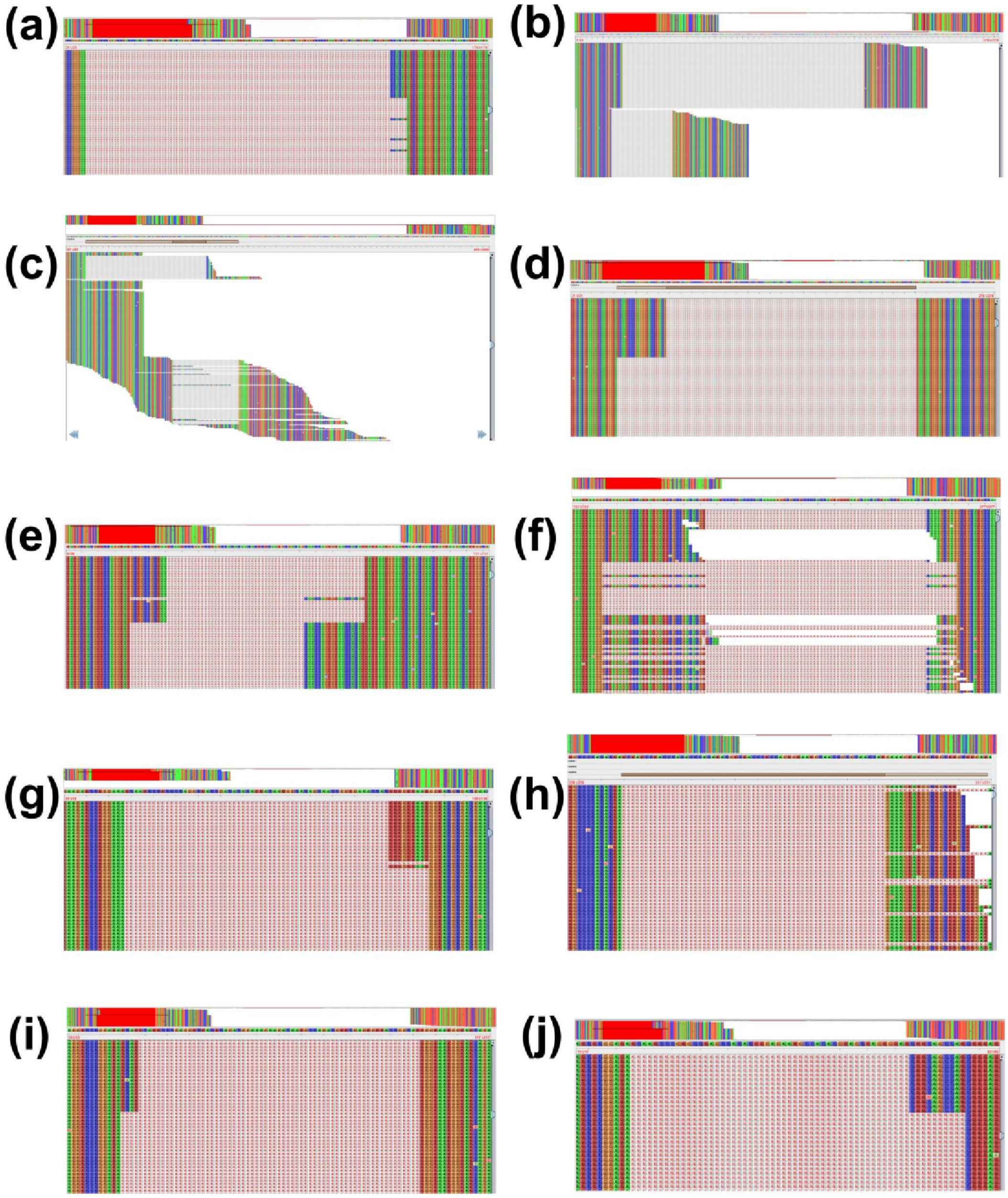

Figure S2 Alternative splicing discovered by aligning sequence reads with reference gDNA. The 10 panels show views with Tablet graphical viewer. (a) First intron of *MbIGYP23*. (b) First intron of *MbIGYP29*. (c) Second intron of *MbIGYP32*. (c) First intron of *MbIGYP36*. (e) First intron of *MbIGYP39*. (f) Second intron of *MbIGYP39*. (g) First intron of *MbIGYP53*. (h) First intron of *MbIGYP56*. (i) First intron of *MbIGYP62*. (j) First intron of *MbIGYP101*.

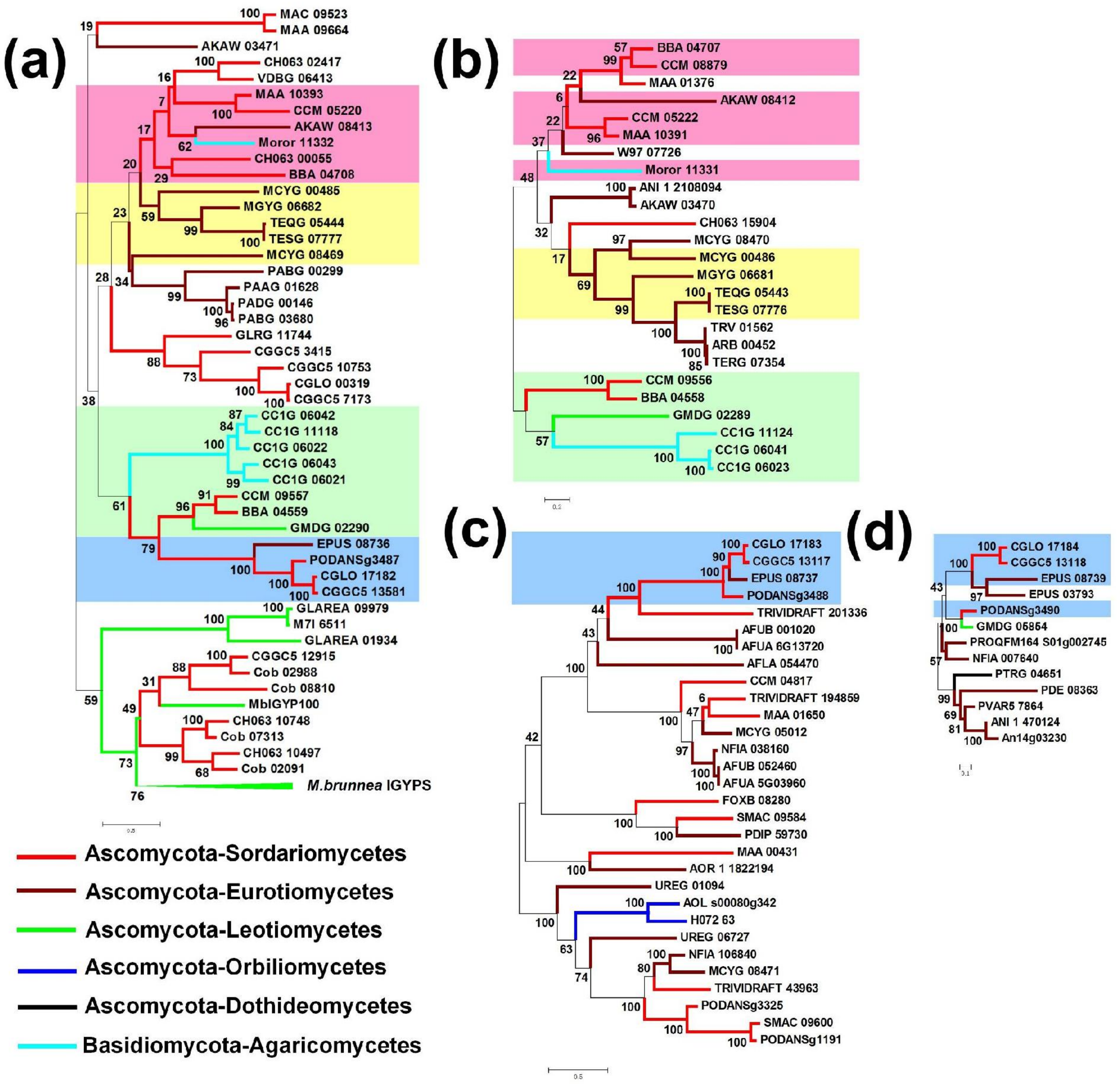

Figure S3 Phylogenies of IGYPs, IGYAP1, ChiC and IGYAP2. Phylogenic trees were inferred by maximum likelihood method with the amino acid sequences (a) Phylogeny of IGYPs. (b) Phylogeny of IGYAP1s. (c) Phylogeny of ChiCs. (d) Phylogeny of IGYAP2s. Colored backgrounds indicate corresponding phylogenetic regions among cluster members.

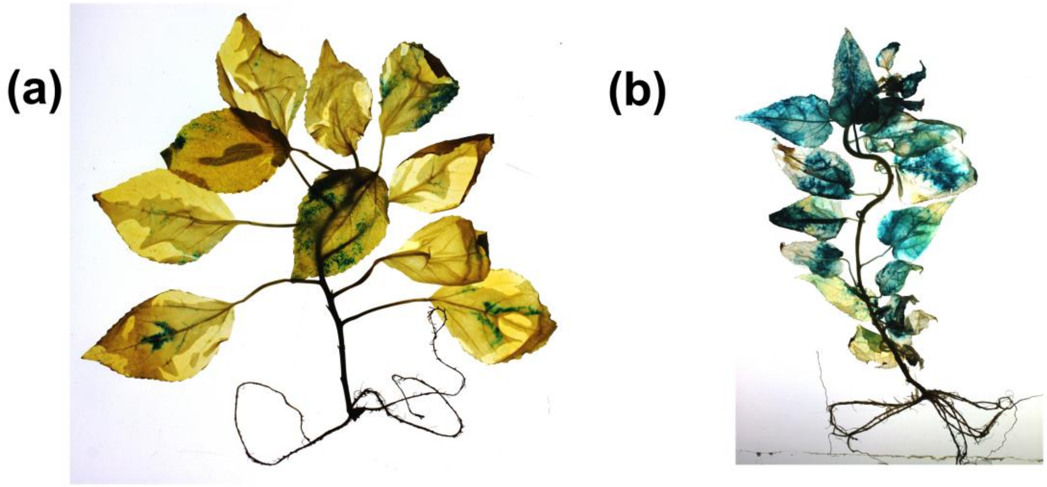

Figure S4 Transient expression of β-glucuronidase (GUS) gene in *P. deltoides* NL895 and *P. tomentosa* (a) AGL1-mediated transient expression of GUS for *P. deltoides* NL895. (b) AGL1-mediated transient expression of GUS for *P. tomentosa*
