# Supplementary material for: Discovery of a novel small secreted protein family with conserved N-terminal IGY motif in Dikarya fungi
Source: BMC Genomics. 2014 Dec 20;15(1):1151. doi: 10.1186/1471-2164-15-1151 (PMC4367982; doi:10.1186/1471-2164-15-1151)
Supplement: Supplementary file 3 — Additional file 3: Statistical data of MbIGYPs RT-PCR-seq results. (PDF 60 KB) [file 12864_2014_6911_MOESM3_ESM.pdf]

	Original number of reads			Number of reads spanned the first intron			Number of reads spanned the second intron		
Gene ID	0 dpi	1 dpi	4 dpi	0 dpi	1 dpi	4 dpi	0 dpi	1 dpi	4 dpi
MbIGYP1	216495	259264	1020374	106833	126807	499221	217	415	1529
MbIGYP2	0	117	131	0	0	0	0	0	0
MbIGYP3	92319	13768	39046	851	196	697	495	151	226
MbIGYP4	3899	25	39590	1934	9	17633	3	0	66
MbIGYP5	51	24813	133996	19	11868	65197	0	53	159
MbIGYP6	19	24213	12899	10	12036	6362	0	98	38
MbIGYP7	36	78591	166507	17	38198	81145	0	21	32
MbIGYP8	198435	282999	178959	808	1952	2139	278	377	427
MbIGYP9	0	0	0	0	0	0	0	0	0
MbIGYP10	175	9	60011	70	3	29035	1	0	62
MbIGYP11	741992	645294	2667082	368726	318016	1305407	379	270	1906
MbIGYP12	2820	219	2052	1170	1	819	30	3	69
MbIGYP13	67150	185791	226480	33461	88914	107846	36	87	116
MbIGYP14	27	32	107838	11	15	50621	0	0	131
MbIGYP15	658	1846	10168	326	817	4486	2	6	13
MbIGYP16	183	133154	206010	83	64086	91647	1	147	194
MbIGYP17	14	31558	20130	5	15266	9797	0	59	49
MbIGYP18	20	43448	54777	5	19391	24536	1	84	111
MbIGYP19	16629	7892	10596	7894	3622	4990	55	61	33
MbIGYP20	114535	39288	547587	56218	19048	268502	153	73	839
MbIGYP21	2	9	33	2	9	31	0	0	0
MbIGYP22	0	0	11	0	0	6	0	0	0

MbIGYP23	22187	221327	64053	10679	108803	31645	86	411	123
MbIGYP24	0	6	170	0	0	88	0	0	1
MbIGYP25	0	5	3	0	0	0	0	0	0
MbIGYP26	30	39	13	0	0	0	0	0	0
MbIGYP27	56850	94027	89799	28268	45712	43504	189	362	103
MbIGYP28	455062	348794	233994	221441	168662	113941	472	692	355
MbIGYP29	71883	227293	95339	36189	110558	46546	210	648	298
MbIGYP30	993	1135	622	362	453	274	95	83	43
MbIGYP31	315922	661695	471641	157867	322918	230901	298	943	606
MbIGYP32	16	4	13149	9	1	6285	0	0	69
MbIGYP33	48	1	85	24	0	36	2	0	0
MbIGYP34	7	2	0	1	0	0	3	1	0
MbIGYP35	27667	59415	76211	13537	28536	36162	103	279	206
MbIGYP36	33	932	4697	17	460	2279	0	9	35
MbIGYP37	10704	270	3717	5283	118	1824	31	0	20
MbIGYP38	690	638	7783	350	306	3787	1	3	27
MbIGYP39	287616	102227	384106	142719	36850	187330	355	277	764
MbIGYP40	137	195	197	70	82	77	0	0	0
MbIGYP41	283742	526681	469212	140734	260243	231992	203	244	276
MbIGYP42	233003	712897	336086	117088	350730	165296	133	833	166
MbIGYP43	14850	46	37473	7203	16	18070	37	1	80
MbIGYP44	528491	407382	304572	262250	198129	147953	746	428	583
MbIGYP45	0	1	2	0	0	0	0	0	0
MbIGYP46	118	2419	10717	52	1079	5069	1	6	35

MbIGYP47	295335	218173	167493	146377	105299	80559	156	145	125
MbIGYP48	1995388	1971975	729460	982852	952695	350510	1419	880	673
MbIGYP49	28060	5044	98	13964	2471	28	87	15	0
MbIGYP50	65156	131308	71613	32096	63766	34579	134	150	189
MbIGYP51	328	272	1087	155	129	530	1	2	7
MbIGYP52	4	1650	5152	2	743	2348	0	1	13
MbIGYP53	3	14592	21233	1	7174	7969	0	21	31
MbIGYP54	55754	1128923	962277	27783	545357	474998	165	677	1378
MbIGYP55	359330	29516	888323	178842	14476	432342	145	23	566
MbIGYP56	11346	457828	190224	5312	222672	91334	74	1079	757
MbIGYP57	3175	0	0	1624	0	0	11	0	0
MbIGYP58	0	0	0	0	0	0	0	0	0
MbIGYP59	7799	3747	6301	3804	1798	2992	19	9	29
MbIGYP60	1155	1494	9488	581	700	4620	5	6	28
MbIGYP61	11239	1010	18729	5691	570	10414	49	7	44
MbIGYP62	154	16	12946	75	7	6223	1	0	54
MbIGYP63	0	0	3	0	0	0	0	0	0
MbIGYP64	536	17	629	214	9	269	16	0	25
MbIGYP65	34	27	107476	18	13	52745	0	0	0
MbIGYP66	65	53	98332	30	6	42985	0	3	461
MbIGYP67	128	374880	189558	57	185828	90512	0	694	620
MbIGYP68	4	12	85	2	0	24	0	0	9
MbIGYP69	0	3	2	0	0	0	0	0	0
MbIGYP70	66	4013	431349	33	1951	209908	1	6	187

MbIGYP71	17	11412	47710	8	5477	22628	0	19	64
MbIGYP72	0	857	11583	0	403	5562	0	0	15
MbIGYP73	278	16500	72374	130	7989	35271	0	29	72
MbIGYP74	7676	1328	66409	3821	652	32461	17	5	106
MbIGYP75	13	127	24186	6	9	11486	0	0	25
MbIGYP76	32	36694	101596	16	17208	52332	0	31	78
MbIGYP77	5	0	3	1	0	0	0	0	0
MbIGYP78	0	763	3839	0	0	3672	0	0	2
MbIGYP79	1	33	839	1	16	507	0	0	2
MbIGYP80	13067	7950	15736	6309	3750	7754	16	6	15
MbIGYP81	4	57	4615	2	12	2259	0	1	8
MbIGYP82	3426	2828	7045	1701	1401	3428	24	13	21
MbIGYP83	20	6218	12777	10	3065	6276	0	17	37
MbIGYP84	1637	10	3569	855	5	1794	20	0	20
MbIGYP85	705	9	904	366	3	469	7	0	6
MbIGYP86	0	989	16752	0	475	8150	0	0	0
MbIGYP87	26	1	3426	11	0	1534	0	0	3
MbIGYP88	122781	247607	5527	26746	118360	2251	358	374	30
MbIGYP89	4924	20746	60	2554	10415	11	9	38	0
MbIGYP90	11	58	22655	6	25	10974	0	1	57
MbIGYP91	96307	217	225621	48023	100	107434	261	5	649
MbIGYP92	25	199	61	12	92	26	0	2	1
MbIGYP93	0	0	0	0	0	0	0	0	0
MbIGYP94	28	1181	39598	14	572	19198	0	3	52

MbIGYP95	15448	25001	77178	7987	12309	37412	22	25	89
MbIGYP96	396	26	260	195	8	134	5	3	4
MbIGYP97	6	1675	4507	3	651	1781	0	4	12
MbIGYP98	34597	20242	7995	17309	9794	3848	68	30	13
MbIGYP99	4579	18	9664	2332	9	4887	8	1	18
MbIGYP100	0	0	0	0	0	0	0	0	0
MbIGYP101	11	9	9134	5	4	4472	0	0	44
MbIGYP102	0	1	0	0	0	0	0	0	0
MbIGYP103	0	8	20	0	4	10	0	0	0
MbIGYP104	1021	1509	473	500	358	106	0	2	0
MbIGYP105	205789	66025	90279	99885	30549	43457	141	49	126
MbIGYP106	0	101	81	0	0	0	0	0	0
MbIGYP107	0	30	12	0	0	0	0	0	0
