# Supplementary material for: Discovery of a novel small secreted protein family with conserved N-terminal IGY motif in Dikarya fungi
Source: BMC Genomics. 2014 Dec 20;15(1):1151. doi: 10.1186/1471-2164-15-1151 (PMC4367982; doi:10.1186/1471-2164-15-1151)
Supplement: Supplementary file 4 — Additional file 4: IGYP, IGYAP1, ChiC and IGYAP2 homologues in Dikarya. (PDF 143 KB) [file 12864_2014_6911_MOESM4_ESM.pdf]

IGYP homologues in Dikarya

Taxonomy and Species	Pathogenicity/reference	Gene ID/access no*.	Signal peptide	N terminal IGY motif	C terminal IP motif	Exon number（sizes bp）
**Ascomycota-Leotiomycetes**						
Marssonina brunnea f. sp. *multigermtubi*	Hemibiotrophic Plant pathogen [1]					
Pseudogymnoascus destructans 20631-21	Bat pathogen [2]	GMDG_02290/GELR06920	yes	AIPIAYRTVGADQA	TQFLIP	3(105/253/263)
Glarea lozoyensis ATCC 20868	N/A [3]	GLAREA_09979/EPE34285	yes	KILIGYRSCSKAEA	PQYLLP	3(147/151/632)
		GLAREA_01934/EPE26022	yes	KILIGYRVVSAEEA	N/A	4(141/88/156/473)
Glarea lozoyensis 74030	N/A [4]	M7I_6511/EHK97727	yes	GDIIGYRTVHDKQA	LQMLIP	4(147/151/176/432)
**Ascomycota-Sordariomycetes**						
Colletotrichum orbiculare MAFF 240422	Hemibiotrophic Plant pathogen [5]	Cob_02091/ENH76548	no	N/A	LQMVIP	2(49/380)

		Cob_07313/ENH84450	yes	RIVIGYRVGEA	LQMLIP	3（99/97/377）
		Cob_02988 /ENH88831	yes	RVLIGFRVVSPEEA	N/A	3(99/149/349)
		Cob_08810/ENH82385	yes	KVLIGFRRVSSAEA	IQMLIP	3(90/149/322)
Colletotrichum gloeosporioides Cg-14	Hemibiotrophic Plant pathogen [6]	CGLO_00319/EQB59303	yes	RSHIGYRIVSKAEA	KQLLIP	3(165/134/361)
		CGLO_17182/EQB44098	yes	RITLGYRTVSKEQA	IQALIP	3(120/134/361)
Colletotrichum gloeosporioides Nara gc5	Hemibiotrophic Plant pathogen [5]	CGGC5_3415/ELA37202	yes	RITLGYRTVSKEQA	KQLLIP	3(126/131/331)
		CGGC5_7173/ ELA32847	yes	RITIGYRTVHPDQA	KQLLIP	3(165/134/361)
		CGGC5_12915/ELA26033	yes	RVLIGFRVVSAKEA	EQLLIP	3(99/50/349)
		CGGC5_13581/ELA25204	no	N/A	IQALIP	2(295/260)

		CGGC5_10753/ELA28676	yes	RITIGYRTVHPDQA	KQLLIP	3(201/134/364)
Colletotrichum higginsianum	Hemibiotrophic Plant pathogen [7]	CH063_10748 /CCF40083	yes	RIVIGYRVVAEAEA	LQMLLP	2(107/548)
		CH063_00055/CCF42999	no	KIIIGYRRVSKEQA	LQMLIP	3（261/140/349）
		CH063_02417/CCF40725	yes	NIIIGYRTVSETQA	LQMVIP	3（129/128/337）
		CH063_10497/CCF39743	yes	REVIGYRVTSKGEA	N/A	3（102/97/95）
Colletotrichum graminicola M1.001	Hemibiotrophic Plant pathogen [7]	GLRG_11744/EFQ36599	yes	RATVGYRTVHPEQA	LQLLIP	1（570）
Metarhizium anisopliae ARSEF 23	Insect pathogen [8]	MAA_10393/EFY94157	yes	NVIIGYRTVAEAQA	QQMVIP	3（129/128/739）
		MAA_09664/EFY94834	yes	RMTIGFRSVGQEEK	QQMLIP	3（126/119/2482）
Metarhizium acridum CQMa 102	Insect pathogen	MAC_09523/EFY	yes	RMTIGFRSVGQEEK	LQLLIP	3（126/119/2473）

	[8]	84421				
Podospora anserina S mat+	N/A [9]	PODANSg3487/XP_001906457	yes	RIHIGYRIVSKEEA	RQALIP	3（120/283/263）
Verticillium alfalfae VaMs.102	Hemibiotrophic Plant pathogen [10]	VDBG_06413/XP_003002851	yes	SVIIGYRTVSAEQG	QQMVVP	3（129/112/317）
Cordyceps militaris CM01	Insect pathogen [11]	CCM_09557/XP_006674753	yes	SVVVGYRTVSAAQA	LQLLIP	3（102/232/332）
		CCM_05220 /XP_006670428	yes	NVIIGYRTVGKHGT	EQMLIP	3（129/155/808）
Beauveria bassiana ARSEF 2860	Insect pathogen [12]	BBA_04559/EJP66619	yes	SIVLGYRTVSAEQA	LQLLIP	3(102/235/263)
		BBA_04708/EJP66215	yes	EIIIGYRRVDPAEA	LQMMIP	3(180/137/1810)
**Ascomycota-Eurotiomycetes**						
Arthroderma otae CBS 113480	Human pathogen [13]	MCYG_00485/XP_002850381	yes	NVIIGYRAVSSEQA	LQLLIP	3（135/131/358）
		MCYG_08469/XP_002842638	yes	SVTIGYRTVSEEQ	DQMLIP	2（132/573）

Arthroderma gypseum CBS 118893	Human pathogen [13]	MGYG_06682/XP_003170693	yes	GVIIGYRTVSDVQA	LQLLIP	3（108/128/352）
Aspergillus kawachii IFO 4308	N/A [14]	AKAW_08413/GAA90299	yes	KVIIGYRTMEKSKA	YQLGIP	3（126/125/331）
		AKAW_03471/GAA85357	yes	EITVGFRRADKTQA	QQMLIVP	3（120/137/334）
Paracoccidioides brasiliensis Pb18	Human pathogen [15]	PADG_00146/EEH43857	yes	KMIIGYMTVSKSIA	VQMSIP	3（165/143/373）
Paracoccidioides brasiliensis Pb03	Human pathogen [15]	PABG_03680/EEH21464	yes	KMIIGYKTVSKSIA	QVQMSIP	3(165/143/373)
		PABG_00299/EEH17736	yes	KMVIGYLAVSAKRA	N/A	7(69/36/66/29/108/133/33)
Paracoccidioides sp. 'lutzii' Pb01	Human pathogen [15]	PAAG_01628/XP_002796620	yes	KMIIGYMTVSKHDR	VQMSIP	3(165/92/373)
Endocarpon pusillum Z07020	N/A [16]	EPUS_08736/ERF72908	yes	KIHVGYRAVHKAEA	IQALIP	3(120/286/266)
Trichophyton equinum CBS 127.97	Human pathogen [13]	TEQG_05444/EGE06442	yes	GDIIGYRTVHDKQA	LQMLIP	3(108/128/349)

Trichophyton tonsurans CBS 112818	Human pathogen [13]	TESG_07777/EGE00433	yes	GDIIGYRTVHDKQA	N/A	3(108/128/139)
**Basidiomycota-Agaricomycetes**						
Coprinopsis cinerea okayama7#130	N/A [17]	CC1G_06042/XP_001829833	yes	TITIGYRMVNKDAA	LQMLIP	3(114/534/225)
		CC1G_06022/XP_001829813	yes	TITIGYRVVPKTVA	LQMVIP	3(114/543/222)
		CC1G_11118/XP_001829848	yes	TITIGYRVVNKDAA	VQMLIP	3(114/556/225)
		CC1G_06043/XP_001829834	yes	TKLIGYRVVPKKVA	VQMLIP	3(114/549/213)
		CC1G_06021 /XP_001829812	yes	AVTIGYRMVSKDTA	VQMLIP	3(114/546/216)
Moniliophthora roreri MCA 2997	Hemibiotrophic plant pathogen [18]	Moror_11332/ESK85283	yes	NIIVGYRTVSQAQA	YQMLIP	2(141/582)

* accession numbers of GenBank

IGYAP1 homologues in Dikarya

Taxonomy and Species	Gene ID/access no*.	Adjacent IGYP gene	Distant IGYP gene
**Ascomycota-Leotiomycetes**			
Pseudogymnoascus destructans 20631-21	GMDG_02289/ELR06919	GMDG_02290	
**Ascomycota-Sordariomycetes**			
Colletotrichum higginsianum	CH063_15904/CCF47559		CH063_10748<br>CH063_00055<br>CH063_02417<br>CH063_10497
Metarhizium anisopliae ARSEF 23	MAA_10391/ EFY94155	MAA_10393	
	MAA_01376/EFZ04302		MAA_09664
Cordyceps militaris CM01	CCM_09556/XP_006674752	CCM_09557	
	CCM_08879/ XP_006674078		
	CCM_05222/ XP_006670430	CCM_05220	
Beauveria bassiana ARSEF 2860	BBA_04558/ EJP66618	BBA_04559	
	BBA_04707/ EJP66214	BBA_04708	
**Ascomycota-Eurotiomycetes**			
Arthroderma otae CBS 113480	MCYG_00486/ EEQ27598	MCYG_00485	
	MCYG_08470/ EEQ35651	MCYG_08469	
Arthroderma gypseum CBS 118893	MGYG_06681/ EFR03684	MGYG_06682	
Arthroderma benhamiae CBS 112371	ARB_00452/ XP_003013267		
Aspergillus kawachii IFO 4308	AKAW_08412/ GAA90298	AKAW_08413	
	AKAW_03470/ GAA85356	AKAW_03471	
Aspergillus niger CBS 513.88	ANI_1_2108094/XP_001394584		
Trichophyton tonsurans CBS 112818	TESG_07776/ EGE00432	TESG_07777	

Trichophyton equinum CBS 127.97	TEQG_05443/ EGE06441	TEQG_05444
Trichophyton rubrum CBS 118892	TERG_07354/ EGD91134	
Coniosporium apollinis CBS 100218	W97_07726/ EON68402	
Trichophyton verrucosum HKI 0517	TRV_01562/ XP_003024283	
**Basidiomycota-Agaricomycetes**		
Coprinopsis cinerea okayama7#130	CC1G_06041/ XP_001829832	CC1G_06042 CC1G_06043
	CC1G_06023/ XP_001829814	CC1G_06021 CC1G_06022
	CC1G_11124/ XP_001829854	CC1G_11118
Moniliophthora roreri MCA 2997	Moror_11331/ ESK85282	Moror_11332

* accession numbers of GenBank

ChiC homologues for phylogenetic analysis

Taxonomy and Species	Gene ID/access no*.	Adjacent IGYP gene	Distant IGYP gene
**Ascomycota-Sordariomycetes**			
Colletotrichum gloeosporioides Cg-14	CGLO_17183/EQB44099	CGLO_17182	
Colletotrichum gloeosporioides Nara gc5	CGGC5_13117/ELA25793	CGGC5_13581	
Endocarpon pusillum Z07020	EPUS_08737/ERF72909	EPUS_08736	
Podospora anserina S mat+	PODANSg3488/XP_001906458	PODANSg3487	
	PODANSg3325/XP_001906297		
	PODANSg1191/ XP_001904174		
Trichoderma virens Gv29-8	TRIVIDRAFT_201336/EHK23200		
Cordyceps militaris CM01	CCM_04817/XP_006670026		CCM_09557 CCM_05220
Trichoderma virens Gv29-8	TRIVIDRAFT_194859/EHK17695		
	TRIVIDRAFT_43963/EHK19035		
Metarhizium anisopliae ARSEF 23	MAA_01650/EFZ02068		MAA_09664
	MAA_00431/EFZ03357		MAA_10393
Fusarium oxysporum Fo5176	FOXB_08280/EGU81130		
Sordaria macrospora k-hell	SMAC_09584/XP_003343643		
	SMAC_09600/XP_003343556		
Penicillium digitatum Pd1	PDIP_59730/EKV10555		
**Ascomycota-Eurotiomycetes**			
Aspergillus fumigatus A1163	AFUB_001020/EDP55411		
	AFUB_052460/EDP51242		
Aspergillus fumigatus Af293	AFUA_6G13720/E		

	AL89210		
	AFUA_5G03960/EAL85910		
Aspergillus flavus NRRL3357	AFLA_054470/EED47392		
Arthroderma otae CBS 113480	MCYG_05012/EEQ32193		MCYG_00485
	MCYG_08471/EEQ35652	MCYG_08469	
Neosartorya fischeri NRRL 181	NFIA_038160/EAW24242		
Aspergillus oryzae RIB40	AOR_1_1822194/XP_001727121		
Uncinocarpus reesii 1704	UREG_01094/EEP76245		
	UREG_06727/EEP81862		
Neosartorya fischeri NRRL 181	NFIA_106840/EAW25195		
**Ascomycota- Orbiliomycetes**			
Arthrobotrys oligospora ATCC 24927	AOL_s00080g342/EGX48217		
Dactylellina haptotyla CBS 200.50	H072_63/EPS45982		

* accession numbers of GenBank

IGYAP2 protein homologues for phylogenetic analysis

Taxonomy and Species	Gene ID/access no*.	Adjacent IGYP gene	Distant IGYP gene
**Ascomycota-Sordariomycetes**			
Podospora anserina S mat+	PODANSg3490/ XP_001906460	PODANSg3487	
Colletotrichum gloeosporioides Cg-14	CGLO_17184/ EQB44100	CGLO_17182	
Colletotrichum gloeosporioides Nara gc5	CGGC5_13118/ ELA25794	CGGC5_13581	
**Ascomycota-Leotiomycetes**			
Pseudogymnoascus destructans 20631-21	GMDG_05854/ ELR02999		GMDG_02290
**Ascomycota-Eurotiomycetes**			
Penicillium oxalicum 114-2	PDE_08363/ EPS33401		
Penicillium roquefort	PROQFM164_S01 g002745/ CDM28934		
Byssochlamys spectabilis No. 5	PVAR5_7864/ GAD99157		
Aspergillus niger CBS 513.88	ANI_1_470124/ XP_001400948		
Aspergillus niger	An14g03230/ CAK46560		
Endocarpon pusillum Z07020	EPUS_03793/ ERF73979		
	EPUS_08739/ ERF72911	EPUS_08736	
Neosartorya fischeri NRRL 181	NFIA_007640/ EAW22088		
**Ascomycota-Dothideomycetes**			
Pyrenophora tritici-repentis Pt-1C-BFP	PTRG_04651/ EDU47558		

* accession numbers of GenBank

Supplemental References:

1. Zhu S, Cao YZ, Jiang C, Tan BY, Wang Z, Feng S, Zhang L, Su XH, Brejova B, Vinar T *et al*: **Sequencing the genome of Marssonina brunnea reveals fungus-poplar co-evolution**. *BMC Genomics* 2012, **13**:382.
2. Chibucos MC, Crabtree J, Nagaraj S, Chaturvedi S, Chaturvedi V: **Draft Genome**

Sequences of Human Pathogenic Fungus Geomyces pannorum Sensu Lato and Bat White Nose Syndrome Pathogen Geomyces (Pseudogymnoascus) destructans. *Genome Announc* 2013, **1**(6).

3. Chen L, Yue Q, Zhang X, Xiang M, Wang C, Li S, Che Y, Ortiz-Lopez FJ, Bills GF, Liu X *et al*: **Genomics-driven discovery of the pneumocandin biosynthetic gene cluster in the fungus Glarea lozoyensis**. *BMC Genomics* 2013, **14**:339.
4. Youssar L, Gruning BA, Erxleben A, Gunther S, Huttel W: **Genome sequence of the fungus Glarea lozoyensis: the first genome sequence of a species from the Helotiaceae family**. *Eukaryot Cell* 2012, **11**(2):250.
5. Gan P, Ikeda K, Irieda H, Narusaka M, O'Connell RJ, Narusaka Y, Takano Y, Kubo Y, Shirasu K: **Comparative genomic and transcriptomic analyses reveal the hemibiotrophic stage shift of Colletotrichum fungi**. *New Phytol* 2013, **197**(4):1236-1249.
6. Alkan N, Meng X, Friedlander G, Reuveni E, Sukno S, Sherman A, Thon M, Fluhr R, Prusky D: **Global aspects of pacC regulation of pathogenicity genes in Colletotrichum gloeosporioides as revealed by transcriptome analysis**. *Mol Plant Microbe Interact* 2013, **26**(11):1345-1358.
7. O'Connell RJ, Thon MR, Hacquard S, Amyotte SG, Kleemann J, Torres MF, Damm U, Buiate EA, Epstein L, Alkan N *et al*: **Lifestyle transitions in plant pathogenic Colletotrichum fungi deciphered by genome and transcriptome analyses**. *Nat Genet* 2012, **44**(9):1060-1065.
8. Gao Q, Jin K, Ying SH, Zhang Y, Xiao G, Shang Y, Duan Z, Hu X, Xie XQ, Zhou G *et al*: **Genome sequencing and comparative transcriptomics of the model entomopathogenic fungi Metarhizium anisopliae and M. acridum**. *PLoS Genet* 2011, **7**(1):e1001264.
9. Espagne E, Lespinet O, Malagnac F, Da Silva C, Jaillon O, Porcel BM, Couloux A, Aury JM, Segurens B, Poulain J *et al*: **The genome sequence of the model ascomycete fungus Podospora anserina**. *Genome Biol* 2008, **9**(5):R77.
10. Klosterman SJ, Subbarao KV, Kang S, Veronese P, Gold SE, Thomma BP, Chen Z, Henrissat B, Lee YH, Park J *et al*: **Comparative genomics yields insights into niche adaptation of plant vascular wilt pathogens**. *PLoS Pathog* 2011, **7**(7):e1002137.
11. Zheng P, Xia Y, Xiao G, Xiong C, Hu X, Zhang S, Zheng H, Huang Y, Zhou Y, Wang S *et al*: **Genome sequence of the insect pathogenic fungus Cordyceps militaris, a valued traditional Chinese medicine**. *Genome Biol* 2011, **12**(11):R116.
12. Xiao G, Ying SH, Zheng P, Wang ZL, Zhang S, Xie XQ, Shang Y, St Leger RJ, Zhao GP, Wang C *et al*: **Genomic perspectives on the evolution of fungal entomopathogenicity in Beauveria bassiana**. *Scientific reports* 2012, **2**:483.
13. Martinez DA, Oliver BG, Graser Y, Goldberg JM, Li W, Martinez-Rossi NM, Monod M, Shelest E, Barton RC, Birch E *et al*: **Comparative genome analysis of Trichophyton rubrum and related dermatophytes reveals candidate genes involved in infection**. *MBio* 2012, **3**(5):e00259-00212.
14. Futagami T, Mori K, Yamashita A, Wada S, Kajiwara Y, Takashita H, Omori T, Takegawa K, Tashiro K, Kuhara S *et al*: **Genome sequence of the white koji mold Aspergillus kawachii IFO 4308, used for brewing the Japanese distilled spirit shochu**. *Eukaryot Cell* 2011, **10**(11):1586-1587.
15. .

16. Wang YY, Liu B, Zhang XY, Zhou QM, Zhang T, Li H, Yu YF, Zhang XL, Hao XY, Wang M *et al*: **Genome characteristics reveal the impact of lichenization on lichen-forming fungus Endocarpon pusillum Hedwig (Verrucariales, Ascomycota)**. *BMC Genomics* 2014, **15**:34.
17. Stajich JE, Wilke SK, Ahren D, Au CH, Birren BW, Borodovsky M, Burns C, Canback B, Casselton LA, Cheng CK *et al*: **Insights into evolution of multicellular fungi from the assembled chromosomes of the mushroom Coprinopsis cinerea (Coprinus cinereus)**. *Proc Natl Acad Sci U S A* 2010, **107**(26):11889-11894.
18. Meinhardt LW, Costa GG, Thomazella DP, Teixeira PJ, Carazzolle MF, Schuster SC, Carlson JE, Guiltinan MJ, Mieczkowski P, Farmer A *et al*: **Genome and secretome analysis of the hemibiotrophic fungal pathogen, Moniliophthora roreri, which causes frosty pod rot disease of cacao: mechanisms of the biotrophic and necrotrophic phases**. *BMC Genomics* 2014, **15**(1):164.
