# Supplementary material for: Discovery of a novel small secreted protein family with conserved N-terminal IGY motif in Dikarya fungi
Source: BMC Genomics. 2014 Dec 20;15(1):1151. doi: 10.1186/1471-2164-15-1151 (PMC4367982; doi:10.1186/1471-2164-15-1151)
Supplement: Supplementary file 5 — Additional file 5: Primers for RT-PCR-seq and real-time RT-PCR. (PDF 65 KB) [file 12864_2014_6911_MOESM5_ESM.pdf]

Primers for RT-PCR-seq and real-time RT-PCR

Gene ID	Forward Primer and reverse primer for RT-PCR-seq	Forward Primer and reverse primer for real-time RT-PCR
MbIGYP1	GATCGGCTACGCAGCACTCA<br>GTAGTGCTCGATGGCCCTCT	
MbIGYP2	GTCATCGGTTATCGAACAGT<br>CCACGATGACTTCTTCGTCT	
MbIGYP3	GAAGAGAAATCATCGGCTAT<br>CTTAGGGTTTTCCATCTTCA	
MbIGYP4	GGACGGATAGTGATCGGCTA<br>CAGTACCACATCGCTACGCT	
MbIGYP5	TTATCGGCTATCGAACAGTC<br>GACTCCGTGCCGCCTGGCTT	
MbIGYP6	AACTATCGGCTATCGATCAG<br>GGTAAGGGCTTCGTTTCCAT	
MbIGYP7	CTCGGTTATCGAACGGTTAG<br>GTCCTCCTGGTCTAGCCACT	
MbIGYP8	GTCATCGGCTACGCAAAGCT<br>TCGGGATCCAGACTTTATCT	
MbIGYP9	ATCATTGGCTATGCAATGAT<br>TCGCTTGAGTTCCATAAAGT	
MbIGYP10	TTGGCTATCGAACAGTTAAC<br>GATTACCTCCATCTCACCAT	
MbIGYP11	ATCGGCTATGCAATGGTTTC<br>GCAAGATGACTTCTTCTTCT	TTTTACCTTCGAGACAAACCTAGC<br>TAACTTCGGATGTAGTGCAAGATG
MbIGYP12	TTATCGGTTATCGAACCGTT<br>CGCTTTCTTCGGGTTCGGCT	
MbIGYP13	GTCATCGGCTATCGAGTCGT<br>ACGGAACCAAGTTGGGTCAT	AGAAGAGTTCCGTCGCTATTTAAC<br>CAAGAGAGCATCATCTTTACGGAA
MbIGYP14	AATTATCGGTTATCAAAAGC<br>AACCTCTGGAAATACAGCCT	
MbIGYP15	AAATTATCGGCTGGCTCACA<br>TCGGGATGGTCCTCTGTGAA	
MbIGYP16	GTTATCGGCTATGCAAAAGT<br>TGAAAGGGTCTTCCGCAAGT	AAAGAAGGGAATTGGTATTGTGCT<br>CAAGACCTCCGCCTCTATATATGA
MbIGYP17	AAATCATCGGGTACCGAACA<br>TAATGCTTTCTCCGGATCTT	
MbIGYP18	CATCGGCTATCGATTAGCTT<br>GGTGAAAAGCGTATTACAGT	
MbIGYP19	AATGGTCATCGGCTATGCAA<br>TAGCGCTTTCTGAGGCTTTC	
MbIGYP20	AGCAAGTTATCGGCTATGCA<br>GGGCTGAGGCAAACCTCTAT	GCTTTTACCTTCGAGATAGACCTG<br>AATAGTCATCTGTCGTTGCTGATC

MbIGYP21	AGAAATCATCGGCTATCGAT<br>CGAGAGGAGTTTTTCGTCTC	
MbIGYP22	GTCATGGGCTATGCAACAGT<br>AATGTACTCTAAAATGACTG	
MbIGYP23	AGGAAATCATCGGCTATGGA<br>GTCTGGCACTTTGGTTTCGA	
MbIGYP24	GATAATCATCGGCTATGCAC<br>TCAATGTAATCCACAATGAC	
MbIGYP25	GAAGAGAAGTCATCGGCTGT<br>GACGCGATATAATTCAGAAC	
MbIGYP26	AGAGTCATCGGCTATGCAAT<br>ATATTGACCAGAGGTATCTC	
MbIGYP27	GTTATCGGCTATCGAACAGT<br>CCCGCGCCACAACACTCTAT	
MbIGYP28	AAGTTATCGGTTATCGAACT<br>TCTCATCCTCCCAGTCCCAT	
MbIGYP29	AAGAGGTTATCGGTTATCGA<br>GCGCAATGCCTCTTCCGCGT	
MbIGYP30	AAGTTATCGGCTATCGAACA<br>GTTGTAGCCCTTGGATGCGA	
MbIGYP31	GAAGTTATCGGTTATCGAAC<br>GCACGCCTATCGACCTGATA	
MbIGYP32	ATCGGCTATCGAACAGTTTC<br>TATGAGAAGCGTAGCGCTCT	
MbIGYP33	AAGTCATCGGCTATGCAAAG<br>CAAGATGACTTCTTCCTCCT	
MbIGYP34	AGAAATCTCGGCTACGGGAT<br>TCTGCAGCTGCCACGGGATA	
MbIGYP35	AGAAACCATCGGCTTTCGAA<br>TCCTACTGCTTTGATGTACT	
MbIGYP36	GAGGAACTAGGGTAACCATC<br>TCGGGGTTTGGGATCATCGA	
MbIGYP37	ACAAATCATCGGCTATCGAA<br>TCCAAGATCTCATCTTCACG	
MbIGYP38	ACTTCTCGGTTATCGAATTG<br>TTCTGACACTATTGTCGTGA	
MbIGYP39	CAGAAGAGCTGATCGGTTAC<br>CGTCCGCGTCGTCGTCAATC	ACAGTTAGGAGCAGGTCTTCATAT<br>TTATTCGCAACACTAGTATACGCC
MbIGYP40	ATTCAGTTATCGGCTATGCA<br>TCCGGGCTCTGGCATCAATG	
MbIGYP41	GTCATCGGCTATGCAAGAGT<br>GTCCACGATGACTTCGTCGC	
MbIGYP42	TCCAGGAATGTTTCGAGGAG<br>TGACGCGAGGTATTCCATGA	

MbIGYP43	GAGAACTCATCGGCTATCGA<br>CCCATCTGATGTATTGCTTA	
MbIGYP44	ATTATCGGCTATCGGACAGT<br>ATATCCAACGAGTCGATGTA	
MbIGYP45	AAAGAATAGTCATCGGCTAT<br>TGAAAAGCGAAGCGCTTTCT	
MbIGYP46	AAGTCATCGGCTATCGAACA<br>CCATTGACTCGATGTAGTTC	
MbIGYP47	TTGTCATCGGCTACGCAACA<br>TGAAGTCCACGATGACATCG	
MbIGYP48	AAAGAGAGGTTATCGCTTAT<br>GATGTAATCCAGAATCCGCT	
MbIGYP49	AAACCATCGCCTATCGAACA<br>TTTGTCGCGATGTACTCCAA	
MbIGYP50	GACAACCATCGGTTATCGAA<br>GCATAAAGTGGAGGTATCTC	
MbIGYP51	GAAGAATTATTGGCTACCGA<br>TGATGTATGACTTGACATCA	
MbIGYP52	AATTATCGCTTACCGAACTG<br>CTTATCTTCGTTTGCGGTCT	
MbIGYP53	AGTCATCGGCTACCGAACAG<br>ATGTATTCCACGGTGAGCTC	
MbIGYP54	AGTTATCGCCTATCGGACTG<br>CTCCAGGATCGCGTCTTCGT	
MbIGYP55	TCATCGGCTATCGAATAGCT<br>TATTCCAGAATGTCTTCTTC	
MbIGYP56	AAATCATCGGCTATCGAACA<br>GCAACGCCTCCTTGGGGTCT	
MbIGYP57	GTGGGACAAGCACGTACTCA<br>TTCGTCGATCTCTTTCGCCT	
MbIGYP58	CTCAAGCTGTAGGAAGCGGA<br>CATAAATCCATTTGCGAAGA	
MbIGYP59	AAAGACGACTTCTCGGTTAT<br>CGTATGAAAAGCGTAATGCT	
MbIGYP60	GGAAGAGAATTTATCGGTTA<br>ATAGTTCAAAAGAGTTTCGT	
MbIGYP61	GATTGTCATCGGCTATCGAA<br>AACCCAGGATCAAATATGGA	
MbIGYP62	AAGAGAGATCATCGCGTATC<br>GCCTCGTTCTTGGACTCGTA	
MbIGYP63	AGAAGTTATCGGGTACCGAA<br>TCGTTTTTAGTCATATAAGT	
MbIGYP64	GTCATCGGCTATGCAGCACT<br>CGCTTTCGTTGGAATAACCA	CAGGATCAATTAGGAAGTGGCTTT<br>TGCCGCTTCTTGAGATTCATAATT

MbIGYP65	AAAGAGAAATTATCGGTTAT<br>TGGATATATTCCAAGAGAGT	
MbIGYP66	AGTCATCGGCTATACAACAG<br>CGCCGGGTGTGACATCCCTA	
MbIGYP67	TATCGGTTATCTACTTGCTA<br>GCCTCATCCGCGTCATCTGA	
MbIGYP68	CGCAATTTTGCTAGCCTCGA<br>AACCGAAATTTGGGATCCTT	
MbIGYP69	GCGATATGGCTAGCTTTAAG<br>ATATAATCCACAATATACTC	
MbIGYP70	TACACGGTCAATAGAAGTTA<br>GGAACCATAATACCTCTCGT	
MbIGYP71	GGCCCTCGAAGACAAATTAT<br>ATACTTTAGAATAGCGTCGT	
MbIGYP72	TCAAGGAATAGTCATCGCCT<br>CGTATCGATGTATCGCAGGA	
MbIGYP73	TCGAATGAAAATCATCGGCT<br>ACGCCCATCGACCTGACGTA	ATGGGCGTTGTGTTAGACTATTTT<br>TCCCATTTGCTTTTATCATCCCAA
MbIGYP74	TTCCTCAAAAGACAAATGGT<br>CACAGAGTACGTCATGATAT	
MbIGYP75	GAAAGTTATCGGCTATGCAA<br>CACTTAAGATAGGCTTCCAG	
MbIGYP76	AGTCATCGGCTATGCAATGA<br>GGGAAATTCCACCAAATCCT	AGATGAAAAGAATCGGCAAAGTCT<br>TTGATTTCTGGCTCCATTTAACGA
MbIGYP77	GACGCCAACTCATCGGCTAT<br>CCTCGGGATCATCTATCGAT	
MbIGYP78	TGGAGTAGAAAGCAAGTCAT<br>AACTGATTCCTCTGCGCCAT	
MbIGYP79	CAGAGTCATCGGCTATGCAA<br>CGATATAATCCGTAATGAGT	
MbIGYP80	CGGAATGTTCCAGCAAAAGA<br>TAATCCATGATGACATCTTC	
MbIGYP81	TAAACCAATAACCATCGGCT<br>ATTTGTTACCCAAGTTGTTG	
MbIGYP82	CAAGCAAAGTCATGGGCTAT<br>CCAGCCCGGACCCATGAGAT	
MbIGYP83	TCCTGCGAGGCAAAGCCATT<br>ATAATGGTGCGCTCGCCTTG	
MbIGYP84	CTCAAGGACGTAACGGTCGA<br>CTTGGTCCCATACGTCTTGT	
MbIGYP85	CAAACCATCAGTCATCGGCT<br>CGCTTTCTTGGAACCCGATG	
MbIGYP86	TAATAACGAACTTATCGGCT<br>GGATATAATCCAAAATGGCT	

MbIGYP87	AGATACCGTCATCGGCTATG<br>CCAAGATAGGTTCTTCGTCT	
MbIGYP88	AAAGACAAGTCATCGGTTAT<br>CTTCTGACACAACATAATTC	
MbIGYP89	AACCAAGTCATTGGCTATGC<br>CGCTTCCTTGGGCTCTGATA	
MbIGYP90	GGCGCAAATTCATTGGCTAT<br>TGGGGTCTTTTATGTATGAT	
MbIGYP91	GCAAGATAAACACACAGTCA<br>TAACTCCGACCTGATATACT	
MbIGYP92	GGCGCAGCAGGATAGTCATC<br>CGCGAAGCGCAGCGCCTTCT	
MbIGYP93	AATAGTTATCGGATATGCAG<br>GGGCTCTCGTAACTTTGCTG	
MbIGYP94	ATGTTTTGGCGCAAAGAAAT<br>CATAGTCGTATTCGCCCTGA	
MbIGYP95	AGACGACAAGAGAATAATCA<br>ATAGTCTGCAATATTTTCCA	
MbIGYP96	GTAGTCATGGCTTATCGAAC<br>TAACAATACTGATTCGATAT	
MbIGYP97	TGCAAGAGAAGCCATCGGCT<br>TGACCTGATGTAATCCAAGA	
MbIGYP98	AGGAAGACAAGTTATCGGCT<br>ATTCGTTCAGGCCCAGCCTC	
MbIGYP99	GAAGAGGACTTATCGCTTAT<br>CTCCGGGTTTTCTGGCGCTA	
MbIGYP101	GTTATCGGCTATGCAGCAGT<br>CGCCTCCGAACCAATATACT	
MbIGYP102	CGAAATTATGGGCTATCGAA<br>ATATATTCTTTGATAACTTC	
MbIGYP103	TACTCATCGGCTATGGTGTA<br>TAATTTGATTTGCTTGTTAG	
MbIGYP104	AGTTATCGGCTATCGAACAG<br>GGAATGAGCATCTGCGTATC	
MbIGYP105	ATGTTAATCGGCTATCGAAC<br>CAGAGGACCTTTTCCTCATA	
MbIGYP106	CTGACGGTGCGGAAAGGGCA<br>AGCTCGATCTCGTCGTCCTT	
MbIGYP107	ATCATCGGCTATCGAACAGT<br>CCAGGACGTTTGCTTCGTCT	
MbNPP1		AACTACAAAGAGATCTTCGCCATC<br>ACTCGAGCAGGTTGAAGTACCAC
MbNPP2		CTATCTGCCATCCGAGAACTCTAC<br>TGAATATTGGTCCAGAACATAGGA

Elongation factor 1-a		GCCCAGGTCATCGTTCTCAACCAC<br>ATCCAATCCTCGCACATCGTAACA
